# A Longitudinal 3D Live-Cell Imaging Platform to Uncover AAV Vector–Host Dynamics at Single-Cell Resolution

**DOI:** 10.3390/ijms27010236

**Published:** 2025-12-25

**Authors:** Marlies Leysen, Nicolas Peredo, Benjamin Pavie, Benjamien Moeyaert, Els Henckaerts

**Affiliations:** 1 Trellis Research Group, Department of Cellular and Molecular Medicine, KU Leuven, B-3000 Leuven, Belgium; marlies.leysen@kuleuven.be (M.L.); benjamien.moeyaert@kuleuven.be (B.M.); 2 VIB BioImaging Core Leuven, VIB Technologies, Center for Brain and Disease Research, B-3000 Leuven, Belgium; nicolas.peredo@kuleuven.be (N.P.); benjamin.pavie@kuleuven.be (B.P.); 3 VIB BioImaging Core Leuven, Department of Neurosciences, KU Leuven, B-3000 Leuven, Belgium; 4 VIB BioImaging Core Ghent, VIB Center for Inflammation Research, B-9052 Ghent, Belgium; 5 Virus–Host Interactions & Therapeutic Approaches Research Group, Department of Microbiology, Immunology and Transplantation, KU Leuven, B-3000 Leuven, Belgium

**Keywords:** live-cell imaging, automated 3D image analysis, viral trafficking, recombinant AAV

## Abstract

Recombinant adeno-associated viral vectors (rAAVs) are the leading gene delivery vehicles in clinical development, yet efficient nuclear delivery remains a major barrier to effective transduction. This limitation is partly due to the incomplete understanding of rAAV’s complex subcellular trafficking dynamics. Here, we establish a longitudinal confocal live-cell imaging workflow that tracks rAAV2 from 4 to 12 h post-transduction, paired with an automated 3D analysis pipeline that quantifies spatiotemporal vector distribution, cytoplasmic trafficking, nuclear accumulation, and transgene expression at single-cell resolution. We use this platform to evaluate the effects of vector dose, cell cycle progression, and the behavior of empty particles. We identify previously undescribed trafficking features associated with high transgene expression. Higher rAAV2 doses enhanced cytoplasmic trafficking and nuclear delivery, while cell cycle progression facilitated both trafficking efficiency and transgene expression. We also characterize empty rAAV2 particles, revealing distinct trafficking patterns and markedly reduced nuclear accumulation compared to genome-containing vectors. By uncovering new bottlenecks in rAAV transduction, this platform provides mechanistic insights and potential strategies to improve AAV-based gene therapy. Its generalizable design further supports broad applicability to other non-enveloped viruses.

## 1. Introduction

Adeno-associated virus is a small (~25 nm), non-pathogenic parvovirus with a non-enveloped icosahedral capsid [1,2]. Its capsid is composed of three viral proteins—VP1, VP2, and VP3—in an approximate 1:1:10 ratio and encloses a linear single-stranded DNA genome. This genome contains two major open reading frames: *rep*, which encodes proteins required for viral replication, and *cap*, which encodes the structural capsid proteins, along with additional regulatory elements [3,4,5]. Recombinant AAV (rAAV) vectors are generated by replacing the native *rep* and *cap* coding sequences with a transgene of interest, enabling delivery of genetic material to host cells through the process of transduction.

Over the past six decades, rAAV has emerged as the prime vector for *in vivo* gene therapy, primarily due to its non-pathogenic nature and capacity for stable, long-term expression across various tissues [6]. The clinical relevance of rAAV-based therapies is evident, with eight already approved treatments and hundreds of active clinical trials [6,7]. However, the therapeutic efficacy of these treatments is constrained by the low transduction efficiency of the vector. This inefficiency necessitates high vector doses, which in turn have been associated with serious adverse events and even patient deaths [8,9,10]. Multiple biological barriers limit efficient *in vivo* rAAV transduction, including limited intracellular trafficking efficiency and poor nuclear entry [6,11,12,13]. Consequently, gaining a better mechanistic understanding of these intracellular processes is crucial for the design of more efficient and safer gene delivery vectors.

The currently available data suggests that (r)AAV vectors enter the cell via a multimodal pathway [11,13,14,15,16]. Following entry, the virions are generally believed to be sequestered into early endosomes before trafficking to the Trans-Golgi network (TGN) [11,13,16]. Multiple cytoplasmic trafficking routes have been proposed, involving early, late, and recycling endosomes [11,13,17,18], as well as cytoskeletal elements such as microtubules and dynein [17,19,20,21,22]. For rAAV2, functional trafficking pathways involving the shuttle protein syntaxin-5 [23] and the multi-serotype receptor AAVR [24] have also been described. Endosomal acidification is believed to be the critical trigger causing conformational changes in the (r)AAV2 capsid proteins VP1/2, which results in the exposure of the viral protein 1 unique (VP1u) region, whose phospholipase A_2_ activity is thought to facilitate subsequent endosomal escape [25,26]. Interestingly, colocalization studies in fixed HeLa cells revealed a dose-dependent trafficking mechanism: at low multiplicities of infection (MOIs), rAAV2 predominantly trafficked through late endosomes, while at higher MOIs, vectors were primarily associated with recycling endosomes, leading to higher transgene expression [18]. This critical link between vector dose and cytoplasmic trafficking efficiency remains poorly characterized, yet it may be a fundamental factor underlying the low *in vivo* transduction efficiency commonly observed with rAAV vectors.

Following escape from the TGN into the cytoplasm, rAAV virions accumulate near the nucleus. Nuclear import is mediated by the exposed nuclear localization signals present on the N-termini of VP1/2 capsid proteins, which facilitate importin-β–dependent nuclear import [27,28]. Using real-time super-resolution fluorescence microscopy, Kelich et al. (2015) [12] demonstrated that rAAV2 particles are imported directly through nuclear pore complexes (NPC). In addition to this conventional pathway, an alternative route has been proposed where AAV may induce localized nuclear envelope breakdown (NEBD). This mechanism, similar to that observed for H1-parvovirus [29] and minute virus of mice [30,31], would allow indirect nuclear entry through transient holes in the nuclear envelope.

Data concerning the kinetics and efficiency of rAAV nuclear entry are notably inconsistent. For rAAV2, the timescale of nuclear entry has been reported to range from as quickly as 15 min [32] to as late as 12 h post-transduction [33,34], although most studies report nuclear accumulation within 1 to 2 h [12,35,36,37]. Furthermore, reported nuclear import efficiencies vary significantly, ranging from as low as ~4% [38] to approximately 20–30% of total cytoplasmic virions [12,38]. Intriguingly, higher vector doses appear to correlate with reduced nuclear import efficiency [38]. Once inside the nucleus, (r)AAV2 accumulates in the nucleolus [39], where it is hypothesized to undergo a stepwise uncoating process leading to the final release of the viral genome [40].

Viruses commonly manipulate the host cell cycle by deregulating checkpoints to promote their replication [41]. For example, wild-type (wt) AAV2’s viral genome and Rep proteins trigger DNA damage responses and interact with cell cycle regulators to induce S- or G2-phase arrest [42,43]. Since recombinant AAV lacks viral genes, its crucial second-strand synthesis and genome conversion steps depend on the host’s S-phase replication machinery [44,45]. This dependency is supported by *in vitro* studies showing that proliferating primary human fibroblast cultures are far more prone to transduction compared to stationary cells [45,46]. Paradoxically, AAV is nonetheless observed to provide stable, long-term gene expression in quiescent or non-dividing cells, including key therapeutic targets like muscle cells [47] and neurons [48]. Although the host cell cycle stage is clearly a major determinant of (r)AAV transduction efficiency *in vitro* and *in vivo*, the specific step(s) in the viral trafficking pathway where cell cycle regulators play a role are currently unknown.

A significant hurdle for rAAV-based gene therapy is the high proportion of capsids that lack the therapeutic transgene. These empty particles can constitute a substantial fraction of the total produced material, depending on the specific production and purification methods [49]. In a clinical setting, empty capsids pose safety concerns, as they can increase host immune responses and increase the risk of unwanted side effects [8,9,50,51]. Furthermore, the competition between empty and full virions may contribute to the overall low *in vivo* transduction efficiency observed in AAV-based therapies. Although it has been reported that empty rAAV2 virions fail to enter the nucleolus [39], remarkably little is known about how their intracellular trafficking pathway differs from that of genome-containing particles.

Over the last few decades, advanced microscopy has greatly aided efforts in understanding viral biology. For instance, live-cell imaging has been instrumental in understanding the viral life cycle of retroviruses, particularly HIV-1 [52]. Advances in super-resolution techniques have pushed the resolution of fluorescence microscopy below the diffraction limit of light and have been applied to the imaging of small viruses such as rAAV [12,53]. In addition, expansion microscopy (ExM), which isotropically enlarges fixed samples, allows for single-virus resolution and has enabled detailed characterization of surface molecular distributions for viruses such as HSV-1 and HIV-1 [54]. More recently, ExM combined with stimulated emission depletion (STED) and single-molecule localization microscopy has visualized SARS-CoV-2 particles, achieving resolutions down to 1 nm [55]. However, super-resolution microscopy remains costly, often requires specialized probes, and is technically demanding, especially for live samples.

In the context of (r)AAV biology, substantial progress has been made in mapping subcellular trafficking using light microscopy (reviewed in Golm et al., 2023 [53]). In spite of these key developments, live-cell imaging studies remain constrained by two major challenges. First, most previously reported imaging approaches examine isolated events, such as cytoplasmic trafficking [17], nuclear import [12], or transgene expression [56], but fail to provide longitudinal kinetic data spanning the entire multi-hour process leading to transduction. Second, most imaging and image analysis approaches are restricted to 2D planes, providing incomplete spatial information about the virus 3D distribution within the host cell. Furthermore, limitations of isolated endpoint studies are underscored by a recent report by Bustamante-Jaramillo et al. (2025) [57], which used time-lapse microscopy to track self-complementary AAV2 genomes and found them colocalized at chromatin rather than nucleoli, contradicting the previously held nucleolar accumulation model [39,40]. Clearly, there is an urgent need for longitudinal, real-time 3D imaging approaches that can track the complete rAAV subcellular trafficking pathway and correlate kinetic subcellular events directly with transgene expression efficiency.

Here, we present a novel live-cell imaging platform to longitudinally track fluorescently labeled rAAV2 particles in cultured cells, including an automated 3D image analysis pipeline. This imaging platform allowed us to quantify viral distribution, cytoplasmic trafficking, and nuclear accumulation, and directly correlate these kinetic events with transgene expression. We assessed how (i) viral dose, (ii) S-phase cell cycle progression, and (iii) capsid genome content (empty versus full) influence both early and later steps of transduction. Our data reveal that higher rAAV2 doses result in significantly higher cytoplasmic trafficking efficiency and nuclear accumulation. Crucially, we establish a direct correlation between cell cycle progression, cytoplasmic trafficking efficiency, and transgene expression. Furthermore, by studying empty rAAV2 vectors in a live-cell context for the first time, we discovered that they exhibit distinct cytoplasmic trafficking and severely impaired nuclear accumulation. Our platform provides critical new mechanistic insights into vector–host dynamics and serves as a modular, broadly applicable tool for studying small, non-enveloped viruses like parvoviruses.

## 2. Results

### 2.1. Real-Time Live-Cell Imaging Workflow

**Figure 1:**
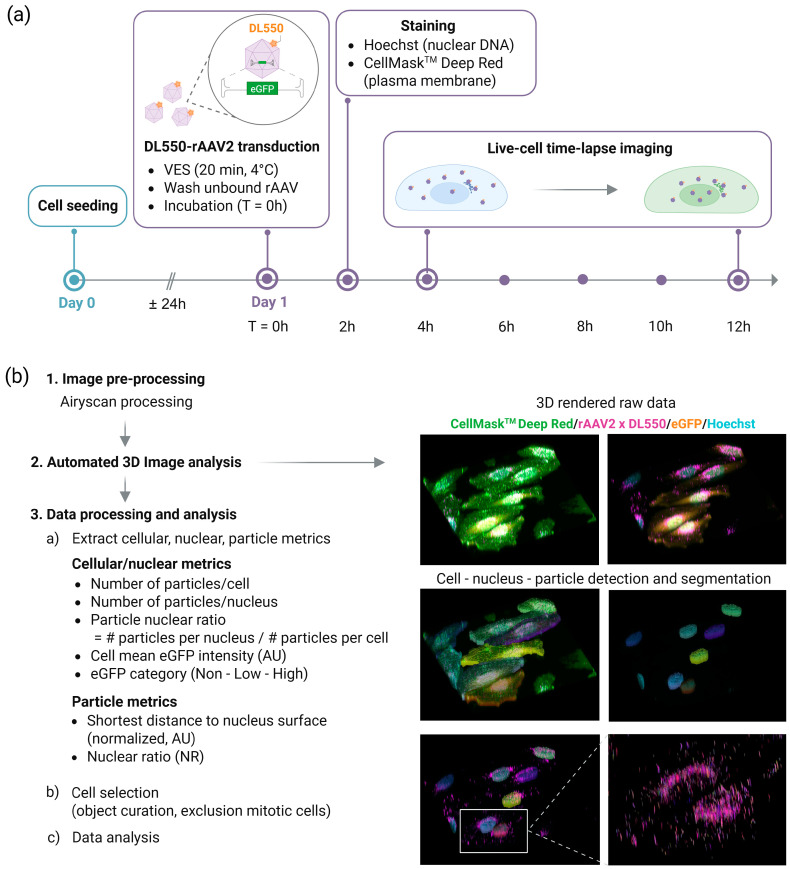
Live-cell confocal imaging and 3D cellular quantification platform. (**a**) Workflow of the live-cell imaging experimental set-up. (**b**) Automated 3D image analysis pipeline allows capturing single-cell level data from hundreds of cells. Cellular, nuclear, and particle metrics were captured. DL550: DyLight^TM^550, VES: vector entry synchronization, AU: arbitrary unit.

Most rAAV trafficking studies rely on fixed cells or live-cell imaging, studying particular phases of the transduction process and thus limiting our understanding of the dynamics of the complete rAAV transduction process. We overcame these limitations by developing a live-cell imaging platform that enables real-time imaging of fluorescently labeled rAAV2 particles from 4 h to 12 h post-transduction, including the read-out of transgene (eGFP) expression (Figure 1a). We first established a robust protocol for labeling rAAV particles with DyLight^TM^ (DL) fluorescent dyes using NHS-ester chemistry (https://doi.org/10.17504/protocols.io.e6nvwqjxwvmk/v1). We confirmed that this labeling has no effect on transduction efficiency (Figure S1). Furthermore, we demonstrated that the labeled particles colocalize with immunofluorescent signals specific to intact AAV2 capsids (Figure S2 [58]). Next, we transduced HeLa cells at an MOI of 5E3 or 5E4 with the labeled vectors. Vector entry synchronization (VES) was accomplished by placing the cells at 4 °C for 20 min, after which the unbound vector was washed away prior to incubating the cells at 37 °C. At 2 h post-VES, cells were stained for nuclear DNA and the cell plasma membrane. A confocal microscope equipped with a CO_2_- and temperature-controlled incubation chamber and Airyscan detector was used to image the cells every 2 h from 4 h to 12 h post-VES (Figure 1a). Images were taken as a z-series with 40 optical sections covering a range of 30.5 µm through the depth of the cell. Control experiments with mock-transduced HeLa cells showed no DL550 signals (Figure S3), indicating that the fluorescent puncta originate directly from the labeled virions.

### 2.2. Three-Dimensional Image Analysis Pipeline

**Figure 2:**
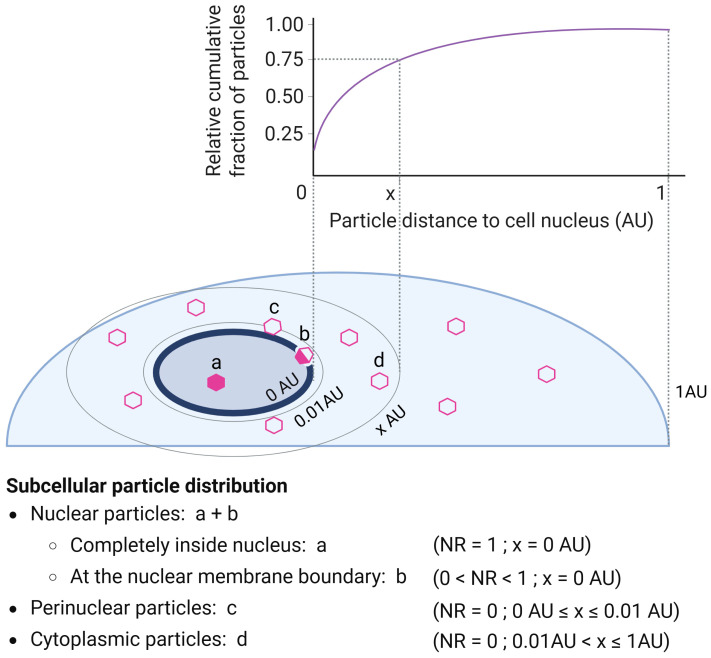
Subcellular distribution and nuclear proximity of rAAV particles. The relative cumulative fraction curve plots the fraction of particles that have accumulated within a distance of x AU or less from the nucleus. For the depicted example, 75% (9 out of 12 particles) of the total cellular particles are accumulated within the distance of x AU or less from the nucleus. Particles detected within cells were classified based on measured nuclear ratio (NR) and distance to the cell nucleus (x) parameters as particles completely inside the nucleus (“a”, NR = 1; x = 0 AU), at the nuclear membrane boundary (“b”, 0 < NR < 1; x = 0 AU), perinuclear particles (“c”, NR = 0; 0 AU ≤ x ≤ 0.01 AU), or cytoplasmic particles (“d”, NR = 0; 0.01 AU < x ≤ 1 AU). The Hoechst DNA marker was used to define the nucleus. The distance of 0.01 AU corresponds to 1% of the maximal distance between the cell nucleus and the plasma membrane and was defined as the perinuclear distance. AU: arbitrary unit, NR: nuclear ratio.

Next, we established an advanced 3D image analysis pipeline capable of processing images of hundreds of individual cells (Figure 1b). Images were first Airyscan processed to increase resolution, enabling viral particle segmentation. Next, an automated 3D image analysis script was run to detect, segment, and track cells, nuclei, and viral particles in 3D-rendered z-stack images (Figure 1b, Video S1 [59]). Cells, nuclei, and particles were correlated at the single-cell level over time. The platform automatically determined key metrics at the cellular, nuclear, and particle level for each of the imaged regions of interest (ROIs), including the number of particles detected per cell/nucleus, particle nuclear ratio, and mean cellular transgene (eGFP) intensity. Furthermore, our script measures—for each detected particle—the shortest distance to the cell nucleus, as well as the nuclear ratio (NR). This NR metric allows discrimination between particles located inside the nucleus, particles at the nuclear membrane boundary, and particles outside the nucleus (Figure 2).

To ensure data quality, output files visualizing cell/nuclei segmentation at different timepoints were manually curated (Figure S4). We specifically excluded cells that underwent mitosis during the imaging period, as the resulting morphological changes (cell rounding and splitting) would bias the particle distance-to-nucleus measurements. As a result, our dataset primarily represents cells in G0, G1, S, and in the early/middle portion of the G2 phase. Furthermore, apoptotic cells and those with improper detection or segmentation were also excluded. A total of 325 cells, containing a total of 312,045 particles, were selected for further data analysis. These cells were distributed across different conditions: Control_no AAV2 (55 cells); AAV2_MOI5E4 (67 cells); AAV2_MOI5E3 (53 cells); AAV2_MOI5E4_S-phase arrest (38 cells); AAV2_MOI5E4_S-phase release (40 cells); AAV2_Empty (72 cells).

**Figure 3:**
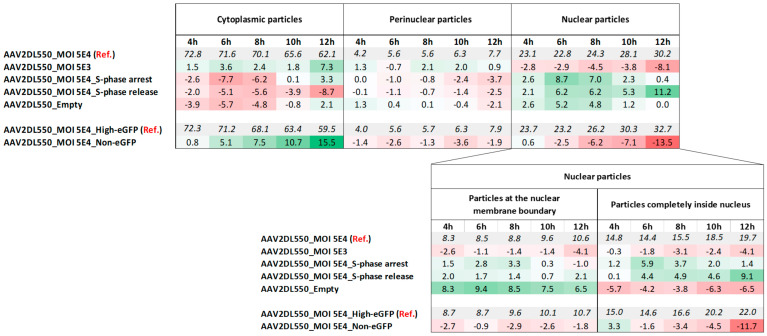
Spatiotemporal and subcellular particle distribution across different conditions. Particles within selected cells of each studied condition and for each timepoint were classified as cytoplasmic particles, perinuclear particles, or nuclear particles (=particles at the nuclear membrane boundary + particles completely inside the nucleus), based on the nuclear ratio (NR) and particle distance to the cell nucleus (x) metrics, as explained in Figure 2. The absolute percentage values of the reference (“Ref.”) conditions (AAV2DL550_MOI 5E4 and AAV2DL550_MOI 5E4_High-eGFP) are depicted in italic values. Differences in absolute percentages compared to the reference conditions are depicted by the red-green color-coded values, indicating lower and higher percentages compared to the reference conditions, respectively. Absolute percentage values are included in Table S1.

To facilitate the interpretation of viral cytoplasmic trafficking kinetics, we plotted the relative cumulative fraction of particles present within a distance (x) from the nucleus for all cells within a condition. Furthermore, the particles were classified as being inside the nucleus, at the nuclear membrane boundary, perinuclear, or cytoplasmic (Figure 2). Calculating these parameters for all particles and cells in a given condition allowed us to quantitatively study the particles’ spatiotemporal distribution in rAAV2-transduced cells (Figure 3, Table S1).

### 2.3. Subcellular Dynamics of rAAV in Cells Exhibiting High Transgene Expression

**Figure 4:**
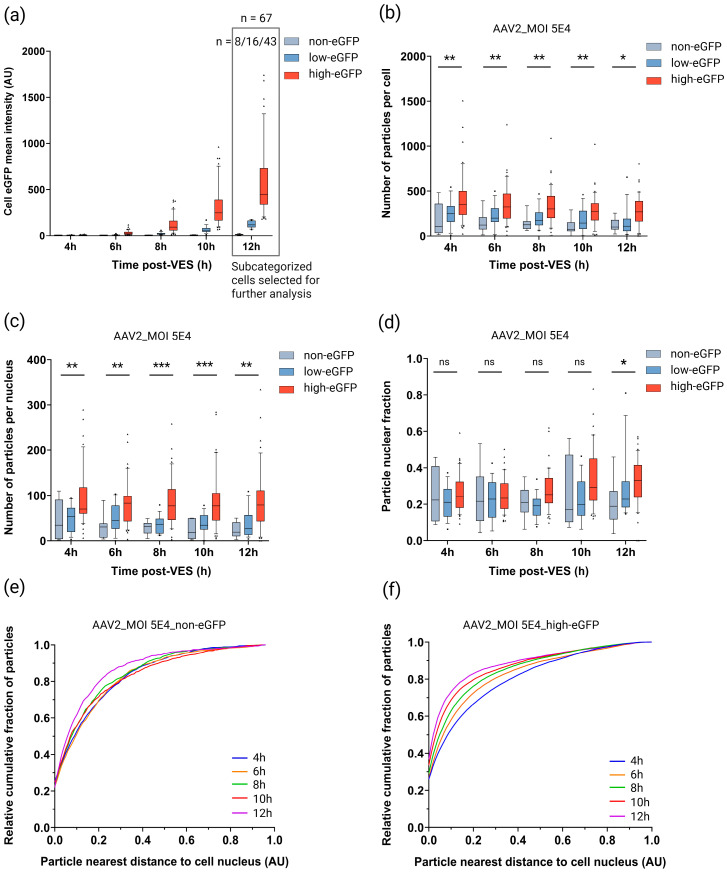
Spatiotemporal subcellular trafficking characteristics of HeLa cells categorized as non-, low- and high-eGFP expressing. HeLa cells were transduced with DL550-labeled rAAV2-eGFP (MOI 5E4), synchronized for vector entry, and imaged in 2 h time intervals from 4 h–12 h post vector entry synchronization (VES). (**a**) Cells were classified as non-, low-, and high-eGFP-expressing based on cell eGFP mean intensity. Subcategorized cells from the final 12 h timepoint were used for further data analysis. n = number of cells analyzed per subcategory. Image-based quantification of (**b**) number of particles per cell, (**c**) number of particles per nucleus, and (**d**) particle nuclear fraction for the subcategorized cells per timepoint are presented. Solid lines, boxes, and whiskers represent medians, lower/upper quartiles, and 10/90-percentile values, respectively. *p*-values were calculated using a 2-tailed Mann–Whitney-U test (ns: *p* > 0.05, *: *p* ≤ 0.05, **: *p* ≤ 0.01, ***: *p* ≤ 0.001). Cytoplasmic viral distribution, represented by the relative cumulative fraction of particles relative to the particle distance to the cell nucleus for (**e**) non-eGFP-expressing and (**f**) high-eGFP-expressing cells over time, is shown. AU: arbitrary unit.

With the aim of understanding the bottlenecks limiting rAAV efficacy, we deployed our live-cell imaging platform to meticulously track the rAAV subcellular trafficking dynamics that correlate with, and potentially lead to, high transgene expression. HeLa cells transduced with DL550-labeled rAAV2 at MOI 5E4 were categorized as non-, low-, and high-eGFP-expressing based on their mean eGFP intensity (see Materials and Methods). Cells classified as high-eGFP showed 39-fold higher eGFP intensity compared to cells classified as non-eGFP-expressing at 12 h post-VES (Figure 4a). For each of the eGFP categories established at the 12 h timepoint, the cell- and particle metrics were extracted, analyzed, and compared across the various timepoints (Figure 4).

Our results showed that the median number of particles per cell was 2.4–3.7 times higher for high-eGFP-expressing cells compared to non-eGFP-expressing cells (Figure 4b). The median number of particles per nucleus for high-eGFP-expressing cells was 2.1–4.3 times higher compared to non-eGFP-classified cells (Figure 4c). Distributions of particle nuclear fractions of both non- and high-eGFP-classified cells were not significantly different for the 4 h, 6 h, 8 h, and 10 h timepoints. However, at 12 h post-VES, a significant 1.8-fold higher median particle nuclear fraction was observed for high-eGFP-expressing compared to non-eGFP-expressing cells (Figure 4d).

In order to compare cytoplasmic trafficking efficiency, we plotted the relative cumulative fraction of particles relative to their nearest distance to the cell nucleus (Figure 4e,f). Our data showed that in non-eGFP expressing cells, the particles’ trafficking toward the nucleus is hampered and less efficient compared to the high-eGFP-expressing cells (Figure 4e,f and Figure S5). The impaired viral trafficking is consistent with an up to 15.5% higher accumulation of cytoplasmic-stalled particles at 12 h post-VES in non- versus high-eGFP-expressing cells (Figure 3, Table S1).

### 2.4. Cytoplasmic Trafficking Efficiency and Nuclear Accumulation Are Dose-Dependent

It was previously reported that rAAV dosing influences the preferred cytoplasmic trafficking pathway and nuclear accumulation efficiency [18,38]. However, this dose-dependent effect has never been studied using longitudinal live imaging with single-cell resolution. Unlike fixed-cell approaches, our platform enabled us to track spatiotemporal dynamics of viral cohorts within the same cells over time. We therefore transduced HeLa cells with DL550-labeled rAAV2 particles at MOI 5E3 and MOI 5E4 and studied viral trafficking dynamics using our live-cell imaging platform.

**Figure 5:**
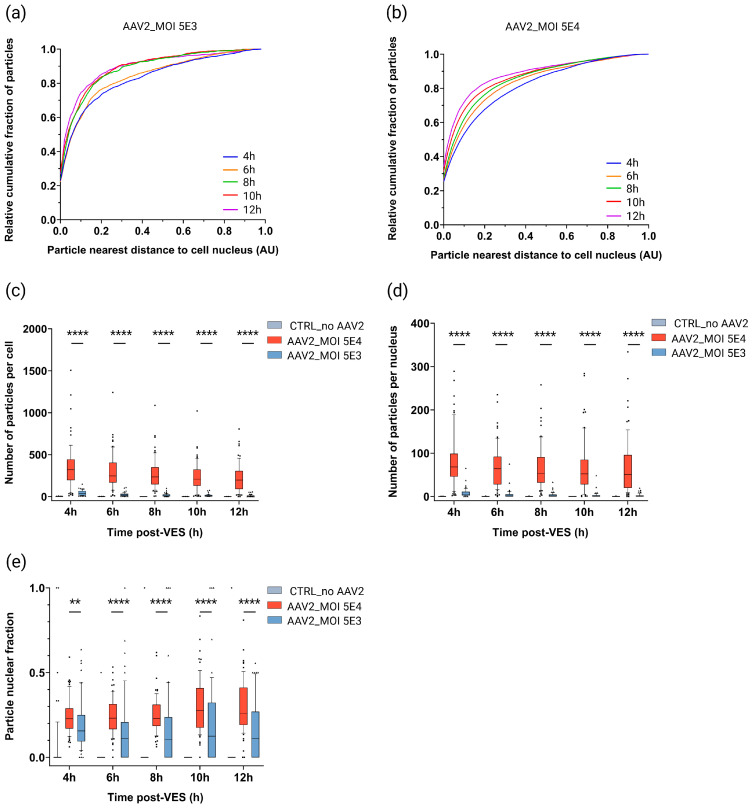
Effect of rAAV dosing on spatiotemporal subcellular trafficking characteristics. HeLa cells were transduced with DL550-labeled rAAV2-eGFP (MOI 5E4 and MOI 5E3), synchronized for vector entry, and imaged in 2 h time intervals from 4 h to 12 h post vector entry synchronization (VES). Cytoplasmic viral distribution represented by the relative cumulative fraction of particles relative to the particle distance to the cell nucleus for (**a**) MOI 5E3 and (**b**) MOI 5E4 transduced HeLa cells over time. AU: arbitrary unit. Image-based quantification of the (**c**) number of particles per cell, (**d**) number of particles per nucleus, and (**e**) particle nuclear fraction for MOI 5E4 (n = 67), MOI 5E3 (n = 53), and mock (n = 55) transduced cells per timepoint. n = number of analyzed cells per condition. Graphs showing a zoomed-in view of the number of particles per cell and number of particles per nucleus for the CTRL_no AAV2 and AAV2 MOI 5E3 conditions can be found in Figure S6c,d, respectively. Solid lines, boxes, and whiskers represent medians, lower/upper quartiles, and 10/90-percentile values, respectively. *p*-values were calculated using a 2-tailed Mann–Whitney-U test (ns: *p* > 0.05, *: *p* ≤ 0.05, **: *p* ≤ 0.01, ***: *p* ≤ 0.001, ****: *p* ≤ 0.0001).

Interestingly, we noticed that at an MOI of 5E3, the cytoplasmic particle distribution reached an equilibrium at 8 h post-VES (Figure 5a and Figure S6a). However, at a 10-fold higher dose (MOI of 5E4), the vectors showed a steady increase in relative cumulative percentages of particles at perinuclear distances over time up to 12 h post-VES (Figure 5b and Figure S6a). At 12 h post-VES, the percentage of cytoplasmic particles was 7.3% higher in cells transduced at MOI 5E3 than at MOI 5E4 (Figure 3, Table S1).

In line with expectations, the median number of particles per cell was 9.1–39.6× higher in MOI 5E4 than in MOI 5E3 transduced cells (Figure 5c). Similarly, the median number of particles per nucleus was higher (13.6–52×) for MOI 5E4 than for MOI 5E3 transduced cells (Figure 5d). Our data also showed a significantly higher (1.5–2.3×) fraction of particles in the nucleus in MOI 5E4 compared to MOI 5E3 transduced cells at all timepoints (Figure 5e). This correlates well with eGFP expression onset: detectable signal between 6 h and 8 h post-VES for MOI 5E4 transduced cells, compared to 10 h–12 h post-VES for MOI 5E3 transduced cells (Figure S6b). The median eGFP expression was, respectively, 8.7, 22.1, and 18.8× higher in MOI 5E4 compared to MOI 5E3 transduced cells at 8 h–10 h–12 h post-VES (Figure S6b). This data further corroborates our observation that cytoplasmic trafficking and nuclear accumulation are dose-dependent.

Furthermore, analysis of the absolute subcellular particle distribution percentages revealed that most particles remained cytoplasmic (62–75%) throughout the 4 to 12 h post-VES time course for both the MOI 5E3 and MOI 5E4 conditions (Table S1). Lower fractions of particles were found perinuclearly (4–9%), and similarly, few particles appeared to be at the nuclear membrane boundary (6–11%) during the imaged time course. A total of 12–20% of the particles were found completely inside the nucleus. Consistent with previous reports [12,38], we found that the fraction of nuclear particles ranged from 20 to 30% (Table S1).

### 2.5. S-Phase Cell Cycle Arrest and Release

It is well established that the host cell cycle stage, and particularly the S-phase, significantly influences rAAV2 transduction efficiency [45,46]. However, the critical link between cell cycle stage, subcellular rAAV2 trafficking, and transduction efficiency has never been analyzed at single-cell resolution. We investigated the effect of S-phase cell cycle arrest and progression on transduction efficiency, as well as on rAAV2 cytoplasmic trafficking efficiency and nuclear accumulation, which are two rate-limiting steps of the transduction process. HeLa cells were blocked in the S-phase using a double thymidine block before transduction with DL550-labeled rAAV2 at MOI 5E4, followed by vector entry synchronization. Cells were either kept in early S-phase or released at the VES timepoint (Figure S7a,b). Control experiments showed that the cells were effectively arrested in the S-phase and subsequently successfully released back into the cell cycle (Figure S8).

#### 2.5.1. Cell Cycle Progression Is Needed for Efficient rAAV2 Cytoplasmic Trafficking Toward the Nucleus and (High) Transgene Expression

**Figure 6:**
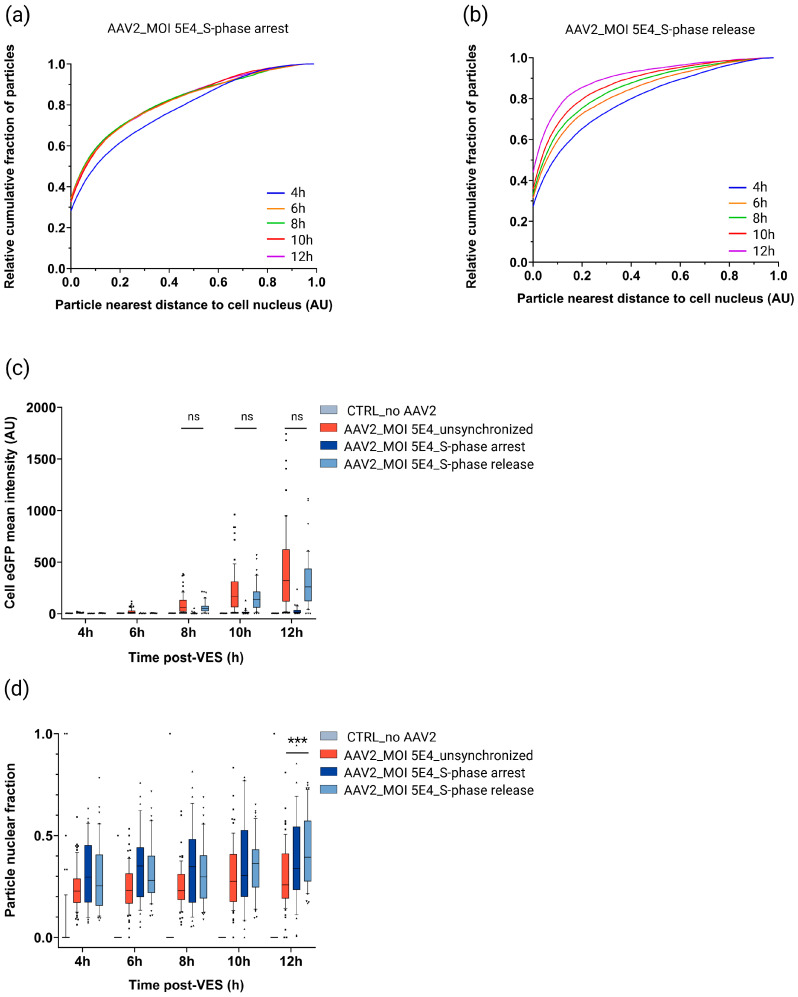
Spatiotemporal subcellular trafficking characteristics of unsynchronized, S-phase-arrested, and S-phase-released HeLa cells. HeLa cells were either synchronized in S-phase or unsynchronized before transduction with DL550-labeled rAAV2-eGFP (MOI 5E4) and vector entry synchronization. Transduced cells were imaged in 2 h time intervals from 4 h to 12 h post vector entry synchronization (VES). Cytoplasmic viral distribution represented by the relative cumulative fraction of particles relative to the particle distance to the cell nucleus for (**a**) S-phase-arrested and (**b**) S-phase-released transduced HeLa cells over time. AU: arbitrary unit. Image-based quantification of the (**c**) eGFP mean intensity, and (**d**) particle nuclear fraction for unsynchronized (n = 67), S-phase-arrested (n = 38), S-phase-released (n = 40), and mock (n = 55) transduced cells per timepoint. n = number of analyzed cells per condition. A zoomed-in view of the cell eGFP mean intensity at the early measured timepoints and the S-phase arrest condition can be found in Figure S9c. Solid lines, boxes, and whiskers represent medians, lower/upper quartiles, and 10/90-percentile values, respectively. *p*-values were calculated using a 2-tailed Mann–Whitney-U test (ns: *p* > 0.05, *: *p* ≤ 0.05, **: *p* ≤ 0.01, ***: *p* ≤ 0.001).

Comparing the cytoplasmic trafficking of viral particles in S-phase-arrested, S-phase-released (Figure 6a,b), and unsynchronized cells (Figure 5b) revealed severely hindered trafficking toward the nucleus for particles in S-phase-arrested cells, but not for S-phase-released and unsynchronized cells (Figure S9a). Furthermore, our data showed that continuous blocking of HeLa cells in S-phase resulted in significantly lower (11.7–20.0–20.8×) cellular mean eGFP fluorescence intensity at 8 h–10 h–12 h timepoints compared to unsynchronized cells (Figure 6c), while unsynchronized cells and S-phase-released cells showed no significant difference in median cell eGFP intensity for these timepoints (Figure 6c). Taken together, these results indicate that progression through the cell cycle (S-phase) is needed for (high) transgene expression.

#### 2.5.2. rAAV Nuclear Import Is Facilitated in S and G2 Cell Cycle Phases

Next, we determined the median fraction of particles in the nucleus and found that it was 1.1–1.5× higher in S-phase-arrested compared to unsynchronized cells (Figure 6d). Intriguingly, the median fraction of particles in the nucleus of S-phase-released cells increased over time, resulting in a significant 1.5-fold higher median fraction of particles in the nucleus at 12 h post-VES compared to unsynchronized cells (Figure 6d). Since S-phase-released cells gradually moved into the G2 phase during live-cell imaging (Figure S8), these observations might suggest that, besides S-phase host cell factors facilitating nuclear import, G2-host cell factors contribute to facilitated nuclear import. Strikingly, despite observing higher median particle nuclear fractions (Figure 6d) and a greater median number of detected nuclear particles (Figure S9b) in the S-phase-arrested cells compared to unsynchronised cells, this did not translate into a corresponding increase in eGFP intensity (Figure 6c and Figure S9c).

### 2.6. Empty Vectors Show Distinct Cytoplasmic Trafficking and Impaired Nuclear Accumulation

Empty vectors may interfere and/or compete with genome-containing vectors during cellular trafficking, yet their involvement in this process remains largely unexplored. We therefore performed longitudinal imaging experiments with empty virions to study their subcellular trafficking in relation to genome-containing particles.

HeLa cells were transduced with 2.08E5 viral particles (vp) per cell of DL550-labeled empty rAAV2 particles, a dosage that was matched to the genome-containing condition (5E4 vg/cell; 2.08E5 vp/cell). Cells were imaged and analyzed using our analysis pipeline. As expected, cells transduced with empty rAAV did not display detectable eGFP expression throughout the entire imaging time course (Figure S10a).

**Figure 7:**
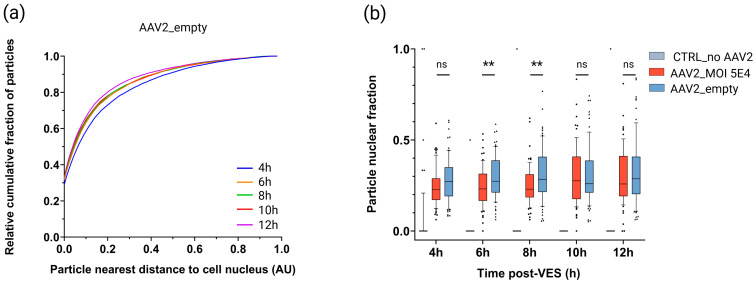
Spatiotemporal subcellular trafficking characteristics of empty rAAV2 particles. HeLa cells were transduced with DL550-labeled rAAV2-eGFP or equivalent amounts of empty DL550-labeled rAAV2 (2.08E5 vp/cell), synchronized for vector entry, and imaged in 2 h time intervals from 4 h to 12 h post vector entry synchronization (VES). (**a**) Cytoplasmic viral distribution represented by the relative cumulative fraction of particles relative to the particle distance to the cell nucleus over time for cells transduced with empty rAAV2. AU: arbitrary unit. (**b**) Image-based quantification of the particle nuclear fraction for genome-containing rAAV2 (MOI 5E4) (n = 67), empty rAAV2 (n = 72), and mock (n = 55) transduced cells per timepoint. n = number of analyzed cells per condition. Solid lines, boxes, and whiskers represent medians, lower/upper quartiles, and 10/90-percentile values, respectively. *p*-values were calculated using a 2-tailed Mann–Whitney-U test (ns: *p* > 0.05, *: *p* ≤ 0.05, **: *p* ≤ 0.01).

Our data demonstrates distinct trafficking toward the nucleus of empty particles (Figure 7a) compared to genome-containing particles (Figure 5b and Figure S10b). In contrast, the particle nuclear fractions of cells transduced with empty vector preps were either not significantly different (4 h, 10 h–12 h) or significantly higher (6 h–8 h) than cells transduced with genome-containing rAAV2 (Figure 7b). Interestingly, we did notice higher absolute percentages (6.5–9.4%) of empty particles at the nuclear membrane boundary over time compared to genome-containing particles (Figure 3, Table S1), although the absolute percentage of empty particles located completely inside the nucleus was 3.8–6.5% lower than that of genome-containing particles (Figure 3, Table S1). This suggests that empty particles are subject to greater retention at the nuclear membrane boundary relative to genome-containing vectors.

## 3. Discussion

Although recombinant adeno-associated virus remains the most widely used vector for *in vivo* gene therapy, its promise is shadowed by a critical limitation: low overall transduction efficiency. The root of this challenge lies, at least partly, in inefficient subcellular trafficking and restricted nuclear delivery. The current cellular and molecular understanding of these barriers is derived primarily from static imaging techniques [53], which fail to capture the dynamic rate-limiting steps [60].

In this work, we developed a 3D live-cell confocal imaging and quantification platform that allows long-term imaging of live cells at single-cell resolution. Key aspects of this platform are (1) a robust labeling protocol, widely applicable to other non-enveloped viruses; (2) an automated, multi-field-of-view, 3D time-lapse imaging setup consisting of an Airyscan confocal microscope in combination with CO_2_- and temperature-controlled incubation; and (3) sophisticated analysis pipelines able to extract cellular and viral particle metrics in three dimensions.

We used this advanced platform to study crucial steps in subcellular trafficking of rAAV vectors by, for the first time, longitudinally imaging cells transduced with rAAV2 viral particles from early timepoints after transduction up to transgene expression. This approach enables us to link rAAV2 cytoplasmic trafficking and nuclear accumulation to transgene expression, yielding new spatiotemporal insights into transduction kinetics. Moreover, our automated quantification enables 3D image analysis of hundreds of cells and offers a more accurate representation of rAAV particle distribution within the cellular environment than 2D methods that depend on arbitrarily selected focal planes.

We found that higher dosing significantly enhanced the efficiency of both cytoplasmic trafficking and subsequent nuclear accumulation. This clearly demonstrates that the early steps of rAAV subcellular trafficking are highly dose-dependent. While the full mechanistic basis for this enhanced efficiency remains to be elucidated, our observation aligns with the findings of Ding et al. (2006) [18], who reported dose-dependent differences in endosomal trafficking. We hypothesize that higher rAAV loads may influence host factors, favoring distinct and more efficient trafficking pathways. The increased nuclear accumulation efficiency may also be connected to the nuclear envelope breakdown hypothesis proposed for several parvoviruses, including AAV. This model suggests that capsids bound to the nuclear pore complex can locally activate mitotic enzymes, transiently disrupting the nuclear envelope to facilitate nuclear entry [29]. A higher vector dose may consequently amplify this effect, inducing a greater number of transient holes that promote rapid vector translocation into the nucleus.

Across all conditions, depending on the vector dose and time post-vector entry synchronization, we measured nuclear particle percentages ranging between 20 and 30%. This range aligns perfectly with previous reports detailing 17–30% nuclear accumulation for rAAV2 [12,38], validating the fidelity of our live-cell imaging platform. More critically, we detected viral particles in the nucleus as early as our first studied timepoint (4 h post-VES). This early entry is followed rapidly by eGFP expression between 6 and 8 h. This tight timeframe suggests that the complete transduction process—including capsid uncoating, second-strand synthesis, transcription, translation, protein folding, and eGFP chromophore maturation—can occur within just 6–8 h.

We next exploited the single-cell resolution of our platform to probe several factors modulating rAAV transduction efficiency. For the first time, we successfully correlated S-phase cell cycle progression and arrest with rAAV cytoplasmic trafficking efficiency, nuclear accumulation, and transgene expression in a longitudinal live-cell imaging setting. Our data provide compelling evidence that active cell cycle progression is essential for efficient cytoplasmic trafficking of viral particles toward the nucleus. Conversely, viral particle cohorts in S-phase-arrested cells exhibited profoundly impaired cytoplasmic trafficking, leading to high percentages of stalled particles in the cytoplasm. Given that rAAV trafficking relies heavily on the host microtubule network organized by the microtubule-organizing center (MTOC) [28], and since MTOC duplication is a hallmark event of the S-phase [61], we speculate that the subsequent alterations in microtubule organization could critically disrupt or limit the efficiency of rAAV transport.

Additionally, our data demonstrates that cells synchronized in the S-phase prior to transduction, whether maintained in the S-phase or released to progress through the S/G2 phase, exhibit higher nuclear import compared to unsynchronized cells. This suggests that host cell factors associated with the S/G2 cell cycle stage facilitate nuclear entry. Furthermore, our findings underscore the requirement for cell cycle progression through the S-phase for achieving (high) transgene expression within the 12 h post-VES window. These findings align with earlier reports showing enhanced transduction in proliferating versus non-dividing cells [45,46]. However, we observed a critical uncoupling: despite higher rAAV2 nuclear accumulation in continuously S-phase-arrested cells compared to unsynchronized cells, these arrested cells displayed significantly lower transgene expression. This key finding indicates that S-phase arrest likely hinders essential post-nuclear import events (e.g., uncoating, genome release, second-strand synthesis, transcription, or translation) necessary for efficient transduction. Although our study did not directly investigate these post-nuclear steps, our findings resonate with those of Sutter et al. (2022) [40], who showed that wild-type AAV2 uncoating depends on nucleolar reorganization during the cell cycle and that G1-phase arrest prevents complete uncoating. Based on our data, it is tempting to speculate that progression through the S/G2 phase (and associated host factors) enhances rAAV2 capsid uncoating. Nevertheless, this progression is not an absolute requirement, as S-phase–arrested cells still manage to express the transgene, albeit at substantially lower levels.

Finally, our study provides the first live-cell imaging evidence detailing the subcellular trafficking of empty rAAV2 particles. We discovered that empty rAAV2 exhibited less efficient cytoplasmic trafficking toward the nucleus and impaired nuclear accumulation compared to genome-containing vector preparations. Mechanistically, these differences in subcellular trafficking likely stem from (but are not exclusively limited to) distinct capsid surface charges [62], altered capsid structure [62,63], or simply the absence of the genomic payload, which can alter interactions needed for efficient subcellular trafficking. The notion that empty capsid impurities could hinder nuclear import of therapeutic genome-containing vectors also underscores the importance of high DNA-containing particle fractions in clinical gene therapy preparations.

In summary, the live-cell imaging platform that we set up is a critical tool in studying the dynamic behavior of viral particles in live cells. We successfully captured several previously unrecognized and critical behaviors of rAAV2. These include the dose-dependency and cell cycle dependency of cytoplasmic trafficking efficiency and nuclear accumulation, as well as the significant impairment of empty rAAV2 particles in entering the nucleus. However, the precise viral and host cell factor interactions driving these observations remain unknown. Our imaging approach positions itself as a powerful, broadly applicable toolkit to start tackling these compelling questions. For instance, a broader dosing range combined with markers for intracellular organelles and/or proteins involved in trafficking (e.g., endosomes, Trans-Golgi network, microtubule network, nuclear envelope, and importin β) could pinpoint the specific host factors driving dose-dependency and its effect on cytoplasmic trafficking, nuclear delivery, and transduction. Additionally, earlier datapoints and shorter time-lapse intervals could further refine our understanding of trafficking kinetics. Further live-cell imaging studies during cell cycle progression and arrest at various cell cycle stages could aid in deciphering the distinct role of the cell cycle in the rAAV subcellular transduction process. Moreover, dual-color live-cell imaging of co-administered empty and full vectors could directly visualize potential competition or interference during the transduction process.

We believe that our labeling, imaging, and analysis platform provides broad utility, which will stimulate further advances in viral research. In addition to its applicability to other rAAV serotypes and cellular models, the platform is readily adaptable to other viruses or fluorescently labeled non-viral structures. Ultimately, we foresee that these types of imaging studies, applied to 3D culture systems such as organoid or *ex vivo* animal or human tissues, will be essential. This leap is critical not only for revealing more unexpected viral characteristics governing viral infection and transduction, but also for successfully translating *in vitro* findings into a therapeutic context.

## 4. Materials and Methods

### 4.1. Cell Culture

HeLa cells (ATCC, Manassas, VA, USA, #93021013) were cultured (37 °C, 5% CO_2_) in phenol red-free Modified Eagle Medium (Thermo Fisher Scientific, Paisley, UK, #51200046) supplemented with 1% GlutaMAX (Thermo Fisher Scientific, Paisley, UK, #35050038), 1% MEM NEAA (Thermo Fisher Scientific, Paisley, UK, #11140050), 100 units/mL of Penicillin and 100 μg/mL of Streptomycin (P/S) (Sigma-Aldrich, Saint Louis, MO, USA), #P0781) (herein called MEM), and 10% Fetal Calf Serum (FCS). Cells were maintained via routine splitting using trypsin-EDTA 0.25% (Sigma-Aldrich, Saint Louis, MO, USA, #T4049) treatment (5 min, 37 °C, 5% CO_2_) when they reached ~80–90% confluency.

### 4.2. Production and Purification of rAAV

#### 4.2.1. Transgene-Containing rAAV

For transgene-containing rAAV vector production, a dual plasmid system was used to transiently transfect HEK 293T/17 cells (ATCC, Manassas, VA, USA, #CRL-11268) using PEI max (Polysciences, Warrington, PA, USA, #24765). The transfer plasmid contained the enhanced Green Fluorescent Protein (eGFP) transgene cassette flanked by ITRs of AAV2 (pKUL-001-CAG-eGFP). The pDG plasmid expressed the AAV2 *Rep* and *Cap* genes and adenovirus 5 helper functions needed to support viral replication [64]. A total of 72 hours post-transfection, cells were harvested. The cell pellet was dissolved in resuspension buffer (50 mM Tris, 150 mM NaCl, 2 mM MgCl_2_, pH 8), before 3 freeze–thaw cycles (each cycle 1 h at −80 °C and 1 h at 37 °C) were applied to produce crude cell lysate and virus-containing supernatant. The cell lysate was treated with Denarase (C-lecta Gmbh, Leipzig, Germany, #20804), centrifuged (15 min, 10,000 rpm), mixed with the supernatant, and filtered through a 0.22 µm membrane filter (VWR, Shanghai, China, #514-0334). rAAV purification was performed on an ÄKTA Pure protein chromatography system (Cytiva, Äkta Avant 25) using a HiTrap Capto AVB column (Cytiva, Uppsala, Sweden, #17372212). An iodixanol step gradient was performed to further purify and polish the rAAV2 vectors. Vectors were buffer-exchanged into Dulbecco’s Phosphate Buffered Saline (DPBS) (Sigma-Aldrich, Saint Louis, MO, USA, #D8537) supplemented with 0.001% Pluronic^®^ F-68 (Thermo Fisher Scientific, Paisley, UK, #24040032) using a 100 kDa MWCO centrifugal filter (Cytiva, Uppsala, Sweden, #GE28-9323-63), then finally filter sterilized and aliquoted for long-term storage at −80 °C.

Viral genome (vg/mL) and viral particle (vp/mL) titers were determined using Droplet Digital PCR (ddPCR) [65] and ELISA (PROGEN), respectively. The genome-containing rAAV2-eGFP prep had a full/total vector ratio of 20.1%.

#### 4.2.2. Empty rAAV

Empty rAAV2 vectors were produced by transfecting VPC 2.0 HEK293 cells (Thermo Fisher Scientific, Seneffe, Belgium, #A49784) in suspension in 1 L shake flasks (Thermo Fisher Scientific, Suzhou, China, #4115-1000) with pDG2 [64] using FectoVir^®^ (Sartorius stedim Biotech GmbH, Goettingen, Germany, #101000022). A total of 72 h post-transfection, 10× lysis buffer (3% Tween-80, 500 mM Tris-(hydroxymethyl)-methylamine, 20 mM MgCl_2_, 2M NaCl (pH 7.5)) and Denarase (C-lecta Gmbh, Leipzig, Germany, #20804) was added to the cells. Flasks were incubated for 2 h at 37 °C, before cells were harvested and centrifuged (30 min, 5500× *g*). The supernatant was filtered, and the cleared lysate was stored at −80 °C until further downstream processing. The latter comprised consecutive filtration of the thawed lysate through a 0.45 µm (VWR, Shanghai, China, #514-0335) and 0.22 µm membrane filter (VWR, Shanghai, China, #514-0334), and further purification on an ÄKTA Pure protein chromatography system (Cytiva, Äkta Avant 25) using a HiTrap Capto AVB column (Cytiva, Uppsala, Sweden, #17372212) and an anion exchange chromatography POROS HQ50 column (Thermo Fischer Scientific, Paisley, UK, #4481315) to separate empty capsids from capsids potentially containing plasmid backbone and/or host cell DNA. After purification, the empty vector was dialyzed into DPBS supplemented with 0.001% Pluronic F-68 (Thermo Fisher Scientific, Paisley, UK, #24040032) using a 100 kDa MWCO Float-A-Lyzer (Merck, Cork, Ireland, #Z727156). Vectors were filter-sterilized and aliquoted for long-term storage at −80 °C.

Viral genome (vg/mL) and viral particle (vp/mL) titers were determined using ddPCR [65] and ELISA (PROGEN), respectively. The empty rAAV2 prep had a full/total vector ratio of 0.0%.

### 4.3. Fluorescent DyLight^TM^550 Labeling of rAAV

AAV particles were labeled with DyLight^TM^ 550 NHS ester dye according to our protocol published at and available from Protocols.io: https://doi.org/10.17504/protocols.io.e6nvwqjxwvmk/v1. The DL550-labeled genome-containing rAAV2-eGFP prep and empty rAAV2 prep had full/total vector ratios of 24.2% and 0.0%, respectively.

### 4.4. Time-Lapse Fluorescent Live-Cell Confocal Imaging

#### 4.4.1. Sample Preparation

HeLa cells were seeded (1.5E4 cells/well) in an ibiTreat 8-well μ-slide (ibidi GmbH, Gräfelfing, Germany, #80806) in phenol red-free MEM10%FCS and incubated overnight. The next day, cells were counted, and DL550-labeled rAAV2 (genome-containing/empty) vector was diluted in cold (4 °C) MEM2%FCS, before adding the vector solution to the cells. The transduced cells were put at 4 °C for 20 min to enable vector entry synchronization (VES) and then washed with cold MEM2%FCS to remove the cell-unbound vector. Next, prewarmed (37 °C) MEM2%FCS media were added to enable vector entry, and cells were incubated (37 °C, 5% CO_2_). This incubation timepoint was marked as T = 0 h. At 2 h post-VES, cells were stained (15 min, 37 °C, 5% CO_2_) using 300 µL/well of a 1 mL staining solution consisting of prewarmed (37 °C) MEM2%FCS supplemented with 1 µL of CellMask^TM^ Deep Red (1:10) (Thermo Fischer Scientific, Paisley, UK, #C10046) as a cell plasma membrane marker, and 10 μL of Hoechst 33342 (Thermo Fischer Scientific, Paisley, UK,#R37605) as a nuclear DNA marker. After staining, cells were washed twice with MEM2%FCS and incubated (37 °C, 5% CO_2_) in MEM2%FCS until confocal image acquisition.

#### 4.4.2. Confocal Image Acquisition

For confocal image acquisition, an inverted Zeiss LSM 880 laser scanning confocal microscope (Carl Zeiss Microscopy GmbH, Oberkochen, Germany) equipped with an Airyscan detector was used in combination with an LD LCI Plan-Apochromat 25×/0.8 Imm Korr DIC M27 objective (NA 0.8). The setup was controlled using ZEN Black software (ZEN 2.3 SP1, Carl Zeiss Microscopy GmbH). The sampling rate was 61.8 nm per pixel in XY, and 762 nm in Z. Images were acquired at 3428 × 3428 pixels with 40 optical sections and a zoom factor of 1.6×. Hoechst was excited with a 405 nm diode laser and detected using a 430–480 nm bandpass (BP) filter, eGFP was excited with a 488 nm Argon laser and detected using a 495–550 nm BP filter, CellMask^TM^ Deep Red Plasma Membrane dye was excited with a 561 nm DPSS laser and detected using a 570–620 nm BP filter, and DyLight^TM^ 550 was excited with a 633 nm HeNe laser and detected using a 645 nm long-pass filter. The images were acquired in Fast Airyscan mode with an 8-bit depth. For each live-cell imaging experiment, 7 regions of interest (ROIs) with ~60–70% confluent HeLa cells were imaged in a time-lapse series of five timepoints, each separated by a 2 h interval, starting at 4 h post-VES. Per studied condition, 3 experimental replicates were performed.

### 4.5. Three-Dimensional Image Analysis

ZEN Black software (Version 2.3) was used to apply Airyscan 3D deconvolution to the images to facilitate viral detection. Time-lapse image files were split into image datasets per timepoint using ZEN BLUE software (Version 2.6).

Image analysis was performed by running the below-mentioned Python (Version 3.10) scripts on either KU Leuven Tier 2 HPC (https://docs.vscentrum.be/leuven/tier2_hardware.html (accessed on 1 December 2025)) or on a local workstation equipped with an Intel Core i7-14700 processor (up to 4.00 GHz), 128 GB DDR5 RAM, and a 1.86 TB SSD for storage. It featured a dedicated NVIDIA RTX A4000 GPU with 16 GB of VRAM and integrated Intel UHD Graphics.

#### 4.5.1. Cell Segmentation and Tracking

Images were opened using AICSImageIO 4.14 [66] and segmented timepoint by timepoint. A maximum intensity projection was applied to each z-stack of the nuclei channel (3) and segmented using CellPose 3.0.10 [67]. In a second step, nuclei were processed with scikit-image 0.20.0 [68]: the images were downscaled using local mean, blurred with a Gaussian filter (σ = 3), automatically thresholded using Otsu’s method, resized, and holes were filled in along the z-dimension. The segmentation results from CellPose were then combined with the threshold-based masks to obtain a more accurate 3D segmentation. Small objects with a volume below 10,000 pixels were excluded. A similar strategy was applied to the cytoplasmic marker channel (0), in association with the corresponding nuclei, to define cells. The 2D labels obtained from CellPose were used to label the 3D binary masks of the cells. Nuclei were then associated with their corresponding cells, and cells without nuclei were excluded. Tracking of nuclei was performed with Trackpy 0.6.4 [69] using a range factor of 400 and a memory of 3. Cells were relabeled according to the nuclei tracks.

#### 4.5.2. Viral Particle Detection

For each timepoint, viral particles were detected in channel 1. Using scikit-image, particle centroids were identified by applying a Laplacian of the Gaussian filter with scikit-image [68]. The channel was then thresholded for multiple values (300, 400, 500, 600, 700, 800), and particles were segmented using a seeded watershed based on the detected centroids. After checking the threshold values on control samples, including mock-transduced cells without rAAV and unlabeled, non-fluorescent rAAV, final particle detection was set to the value of 600.

#### 4.5.3. Cell, Particle, and Nucleus Measurements

Measurements were performed with scikit-image to extract multiple features per cell, including shape and intensity features for cells, nuclei, and cytoplasm; the number of particles per nucleus and per cytoplasm; and particle distances to the cell and nuclear membranes. A nuclei ratio metric was included that calculates, for each of the detected particles, the ratio of the volume of the particle that is completely inside the nucleus over the total volume of the particle, providing information about (in)complete nuclear accumulation.

#### 4.5.4. Data Processing and Analysis

Image-derived measurements were processed using a custom analysis pipeline implemented in Python (Version 3.9). Individual measurement files corresponding to each region of interest and timepoint were combined into a single dataset, and experimental metadata such as condition, timepoint, and ROI were extracted and standardized. Timepoints were categorized as 4 h, 6 h, 8 h, 10 h, and 12 h post-VES, and experimental conditions were harmonized across replicates.

For cell- and nucleus-level analysis, segmentation outputs were merged to obtain cell and nuclear volumes, the number of viral particles per cell and per nucleus, and mean eGFP intensity per cell. Using this data, the nuclear fraction of viral particles was calculated as the ratio of nuclear to total cellular particles. Additionally, per studied condition, cells were categorized as “non-”, “low-” or “high-” eGFP-expressing via percentile thresholding based on mean eGFP intensity values of all cells over all timepoints of that condition (non-eGFP (0–50th percentile), low-eGFP (50th–75th percentile), and high-eGFP (75th–100th). For particle-level analysis, the shortest distance from each viral particle to the nuclear boundary was calculated and normalized for cell size by dividing it by the maximum nucleus-to-membrane distance within the same cell. To ensure data quality, only accurately segmented cells were included in the analysis. Cell selection was performed by an expert based on segmentation accuracy and related quality parameters, excluding any cells with poor segmentation, and cells (going) in mitosis, since these cells can bias particle-distance-to-the-nucleus measurements.

Processed datasets were used to generate descriptive and comparative visualizations. Graphics were performed using GraphPad Prism 9 (Boston, MA, USA). Boxplots were employed to compare cellular/nuclear particle counts, nuclear particle fractions, and cellular eGFP transgene expression across timepoints and conditions. To explore spatial aspects of particle cytoplasmic trafficking, cumulative distribution plots were generated to illustrate the distribution of distances between viral particles and the nuclear membrane at different timepoints. Detected particles within cells were, based on measured nuclei ratio (NR) and distance to the cell nucleus (x) parameters, classified as particles completely inside the nucleus (NR = 1; x = 0 AU), particles at the nuclear membrane boundary (0 < NR < 1; x = 0 AU), perinuclear particles (NR = 0; 0 AU ≤ x ≤ 0.01 AU), or cytoplasmic particles (NR = 0; 0.01 AU < x ≤ 1 AU).

### 4.6. HeLa S-Phase Cell Cycle Arrest and Release

Flow cytometry analysis was performed to check for cell cycle synchronization and progression. Briefly, HeLa cells were seeded in a 6-well plate (4.8E4 cells/well in MEM10%FCS medium) in parallel with the cells used for live-cell imaging (8-well µ-slide; 1.5E4 cells/well in MEM10%FCS medium) and synchronized in the S-phase by using a double thymidine block (2 mM) (Figure S7a,b). Cells were either continuously blocked in the S-phase (Figure S7a) or released from S-phase arrest (Figure S7b) and fixed (70% ethanol) at various timepoints post-VES (2 h–3 h post-VES (Figure S7a), as well as 15 min and 2 h15 min post-VES (Figure S7b). Cells were stored at −20 °C and analyzed using flow cytometry via propidium iodide staining for cell cycle classification. Flow cytometry experiments were, in correlation with live-cell imaging experiments, performed in triplicate.

Cells seeded for live-cell imaging were counted after thymidine blocking (3 out of 6 seeded wells) to calculate the amount of rAAV needed for transduction (Figure S7a,b). Cells in the remaining wells were visually checked for confluency (~60–70%) before one well was selected for transduction, VES, and washing, with media containing 2 mM thymidine for continuous S-phase arrest (Figure S7a) or no thymidine for S-phase-released cells (Figure S7b).

### 4.7. Statistical Analysis

Statistical analyses were performed using GraphPad Prism 9 (Boston, MA, USA). Distribution assumptions of the data were checked visually. The nonparametric Mann–Whitney-U test (two-tailed, α = 0.05 significance level) was used to statistically compare two unpaired groups of data regarding the intended parameters (cell eGFP mean intensity, number of particles per cell, number of particles per nucleus, particle nuclear fraction), unless other statistical tests were mentioned.

## Data Availability

The original contributions presented in the study are included in the article/Supplementary Materials. The raw microscopy data will be made available by the authors upon reasonable request. Image analysis scripts are available from GitHub (https://github.com/vib-bic-projects/2025_10_Marlies_Leysen_rAAV (accessed on 1 December 2025)).

## Supplementary Materials

The following supporting information can be downloaded at: https://www.mdpi.com/article/10.3390/ijms27010236/s1.

## Abbreviations

The following abbreviations are used in this manuscript:

rAAV	Recombinant adeno-associated virus
Rep	Replication
Cap	Capsid
TGN	Trans-Golgi network
VP1u	Viral protein 1 unique region
MOI	Multiplicity of infection
NPC	Nuclear pore complex
NEBD	Nuclear envelope breakdown
Wt	Wild-type
eGFP	Enhanced green fluorescent protein
DL	DyLight^TM^
VES	Vector entry synchronization
ROI	Region of interest
NR	Nuclear ratio
AU	Arbitrary unit
ExM	Expansion microscopy
STED	Stimulation emission depletion
MTOC	Microtubule-organizing center
DPBS	Dulbecco’s Phosphate Buffered Saline
ddPCR	Droplet digital polymerase chain reaction
