# Supplementary material for: A Longitudinal 3D Live-Cell Imaging Platform to Uncover AAV Vector–Host Dynamics at Single-Cell Resolution
Source: Int J Mol Sci. 2025 Dec 25;27(1):236. doi: 10.3390/ijms27010236 (PMC12785796; doi:10.3390/ijms27010236)
Supplement: Supplementary file 1 [file ijms-27-00236-s001.zip › ijms-4042811-supplementary proofed.pdf]

Supplementary Materials

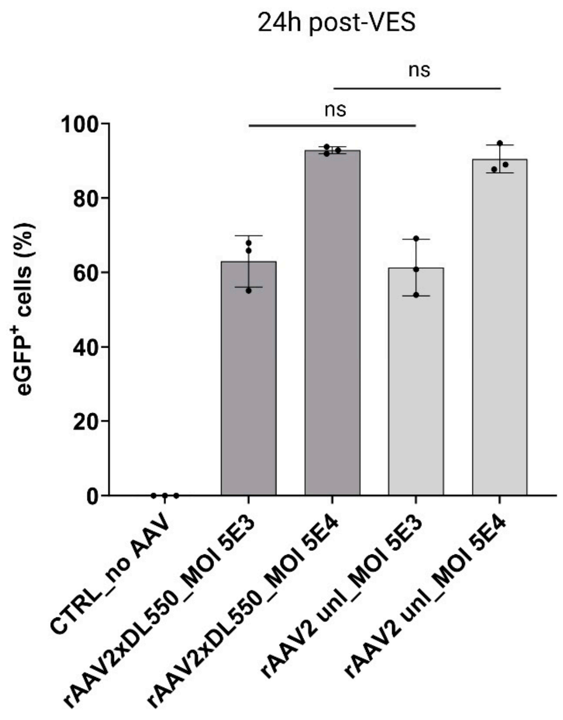

Figure S1. DL550 labeling does not affect rAAV2 transduction efficiency in HeLa cells.
HeLa cells were mock-transduced with DPBS (CTRL) or transduced with DL550-labeled and unlabeled (unl) rAAV2 at MOI 5E3 and MOI 5E4. After transduction, cells were synchronized for vector entry (VES, 20 min at 4°C), washed to remove unbound vector, and incubated (37°C, 5% CO_2). At 24h post-VES, cells were fixed and analyzed by flow cytometry for eGFP transgene expression. Data represented as mean ± SD of three independent experiments. P-values were calculated using an unpaired Student's t-test. (*ns: p > 0.05*). DPBS: Dulbecco's Phosphate-Buffered Saline, DL550: DyLight^{TM}550, MOI: multiplicity of infection.

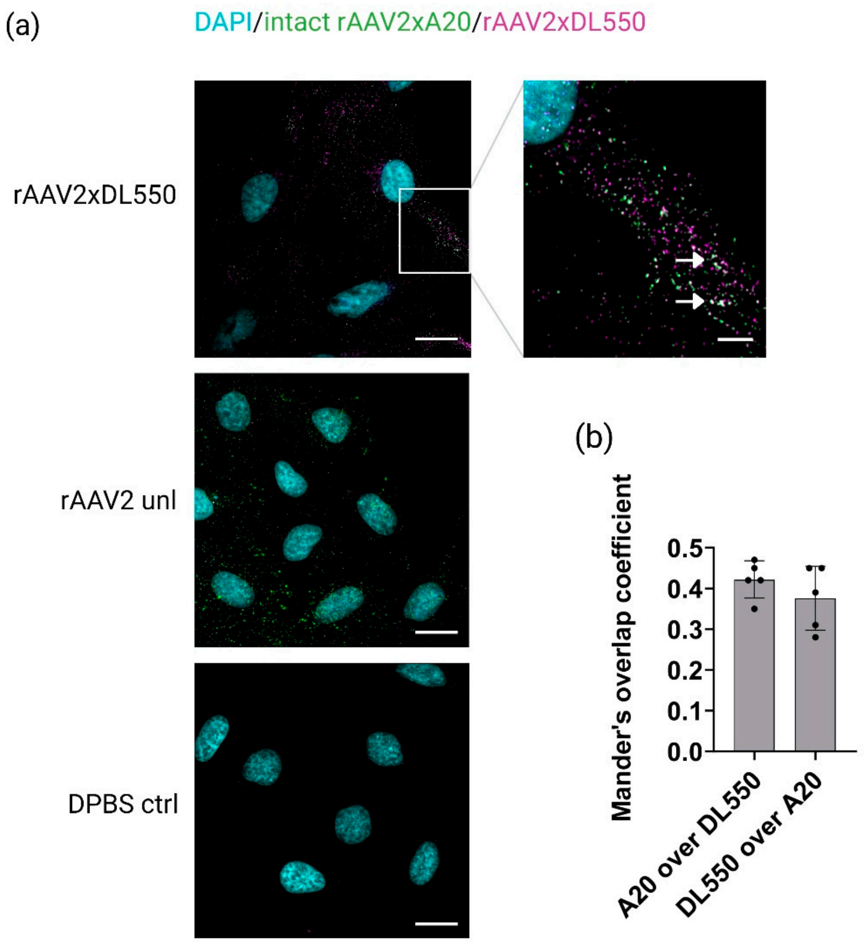

Figure S2. Colocalization of DL550-labeled rAAV2 and intact AAV2 capsids in HeLa cells.
(**a**) HeLa cells were transduced with DL550-labeled or unlabeled (unl) rAAV2 at MOI 5E4 or mock-transduced (DPBS ctrl), synchronized for vector entry (4°C, 20 min), washed to remove unbound vector and incubated (37°C, 5% CO_2). After 30 min, cells were fixed and stained for immunofluorescence with an antibody (A20, Progen, #61055) to detect intact AAV2 capsids (green). Nuclei were stained with DAPI (blue). Cells were imaged as midsections using a confocal laser scanning microscope equipped with an Airyscan detector and a 40x oil immersion objective. The scale bars represent 20 µm (images left) and 5 µm (detail image). Cells transduced with the unlabeled rAAV2 showed only A20 signals. In cells transduced with DL550-labeled rAAV2, A20 signals colocalized with DL550 signals (indicated arrows). DPBS: Dulbecco's Phosphate-Buffered Saline, DL550: DyLight^{TM}550, MOI: multiplicity of infection. (**b**) The colocalization between DL550-labeled rAAV2 and A20 intact AAV2 signals was quantified using Manders' overlap coefficient using the JACoP plugin within ImageJ [58]. This coefficient measures the fraction of the intensity of one channel (DL550) that overlaps with the other channel (A20). A value close to 1 indicates a high degree of overlap, while a value of 0 suggests no overlap. Bars represent mean ± SD of 5 analyzed images.

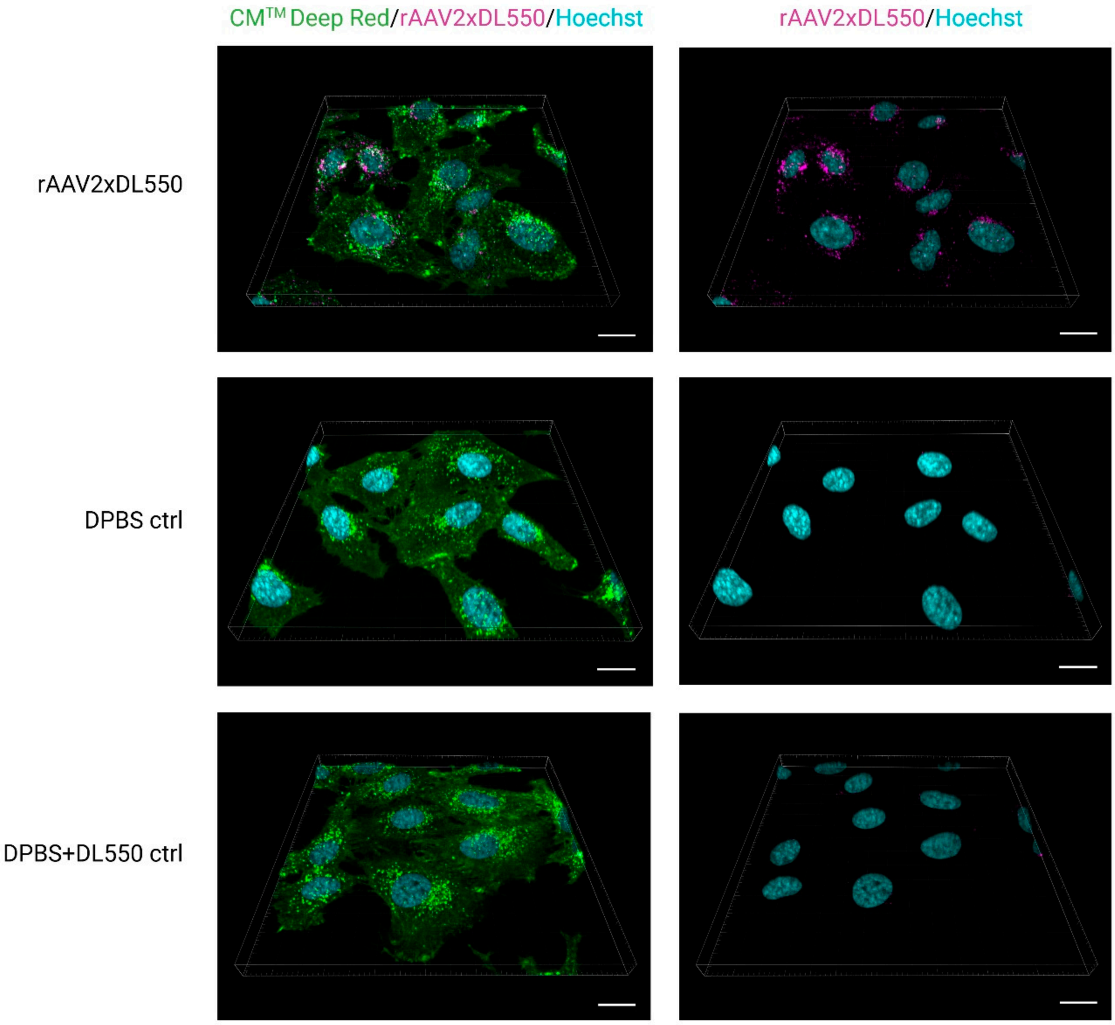

Figure S3. Live-cell imaging of DL550-labeled rAAV2 particles with negligible background signal.
Hela cells were transduced with DL550-labeled rAAV2 at MOI 1E4, or mock transduced with DPBS or DL550-DPBS mixture. Cells were synchronized for vector entry (VES, 4°C, 20 min), washed and incubated (37°C, 5% CO_2). At 2 hours post-VES, cells were stained with CellMask™ Deep Red and Hoechst to label the cell plasma membrane and nucleus, respectively. Cells were imaged using a confocal laser scanning microscope equipped with an Airyscan detector and a 40x oil immersion objective. 3D-rendered z-stack images were visualized using Imaris Viewer software (Version 10.2.0). The scalebars represent 20 µm. DPBS: Dulbecco's Phosphate-Buffered Saline, DL550: DyLight™550, MOI: multiplicity of infection.

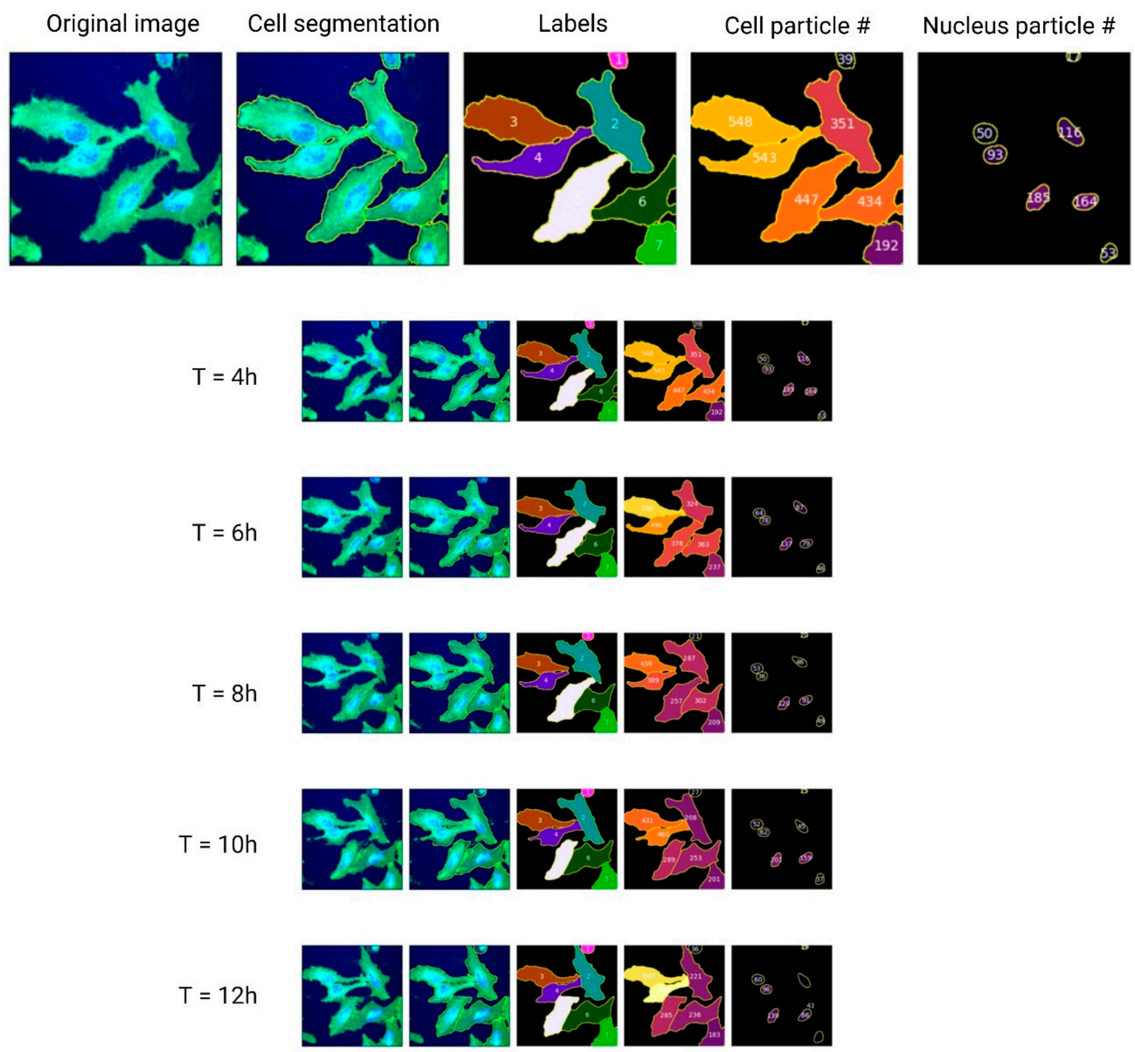

Figure S4. Data analysis output files allow for the visualization of cell and nucleus segmentation as well as the quantification of detected particles in the cell and nucleus over time.

Our data analysis creates, for each of the regions of interest, output files containing a maximum intensity projection of the original (z-stack) images, with marked cell segmentation, associated cell label numbers and number of particles detected in the segmented cell/nucleus. A color scale was integrated to allow easy visual interpretation.

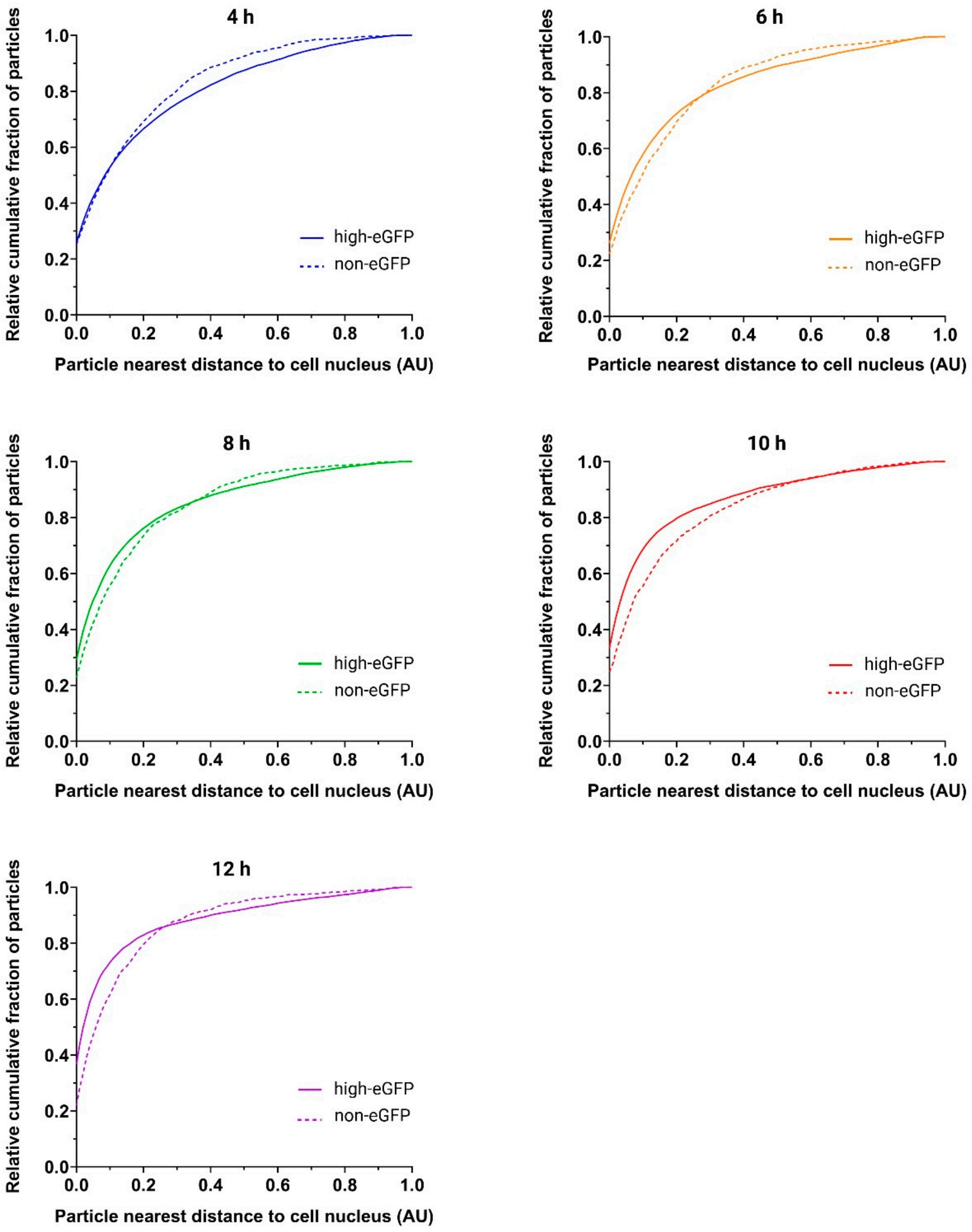

Figure S5. Spatiotemporal cytoplasmic particle distribution in non- and high-eGFP-expressing cells transduced with DL550-labeled rAAV2 at MOI5E4. Graphs represent the relative cumulative fraction of particles relative to the particle nearest distance to the cell nucleus for non-eGFP and high-eGFP-expressing cells over time. DL550: DyLight^{TM}550, MOI: multiplicity of infection, AU: arbitrary units.

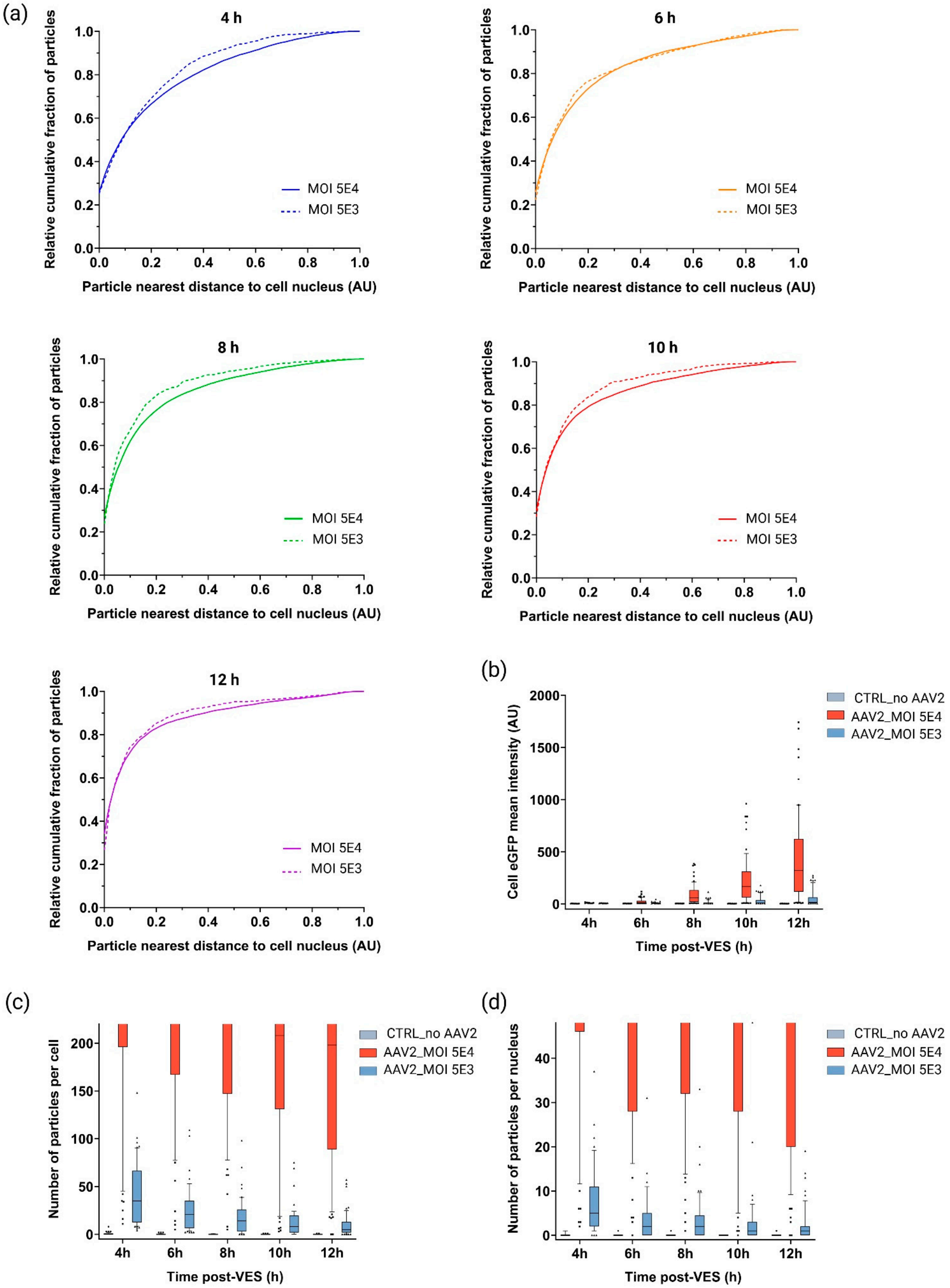

(a)
4 h
6 h
8 h
10 h
12 h
Relative cumulative fraction of particles
Particle nearest distance to cell nucleus (AU)
MOI 5E4
MOI 5E3
(b)
Cell eGFP mean intensity (AU)
Time post-VES (h)
CTRL_no AAV2
AAV2_MOI 5E4
AAV2_MOI 5E3
(c)
Number of particles per cell
Time post-VES (h)
(d)
Number of particles per nucleus
Time post-VES (h)
4h
6h
8h
10h
12h

Figure S6. Effect of rAAV dosing on spatiotemporal subcellular trafficking characteristics.

(**a**) Cytoplasmic particle distribution from cells transduced with DL550-labeled rAAV2 at MOI 5E3 and MOI 5E4. Graphs represent the relative cumulative fraction of particles relative to the particle nearest distance to the cell nucleus. (**b**) Image-based quantification of eGFP mean intensity for mock (n = 55), MOI 5E4 (n= 67) and MOI 5E3 (n= 53) transduced cells per timepoint. Graphs with zoomed-in y axis for the number of detected particles per cell (**c**) and per nucleus (**d**) (graphs related to Figure 5c & d respectively). n= number of analyzed cells per condition. Solid lines, boxes and whiskers represent median, lower/upper quartiles and 10/90-percentile values, respectively. DL550: DyLight™550, MOI: multiplicity of infection, AU: arbitrary units.

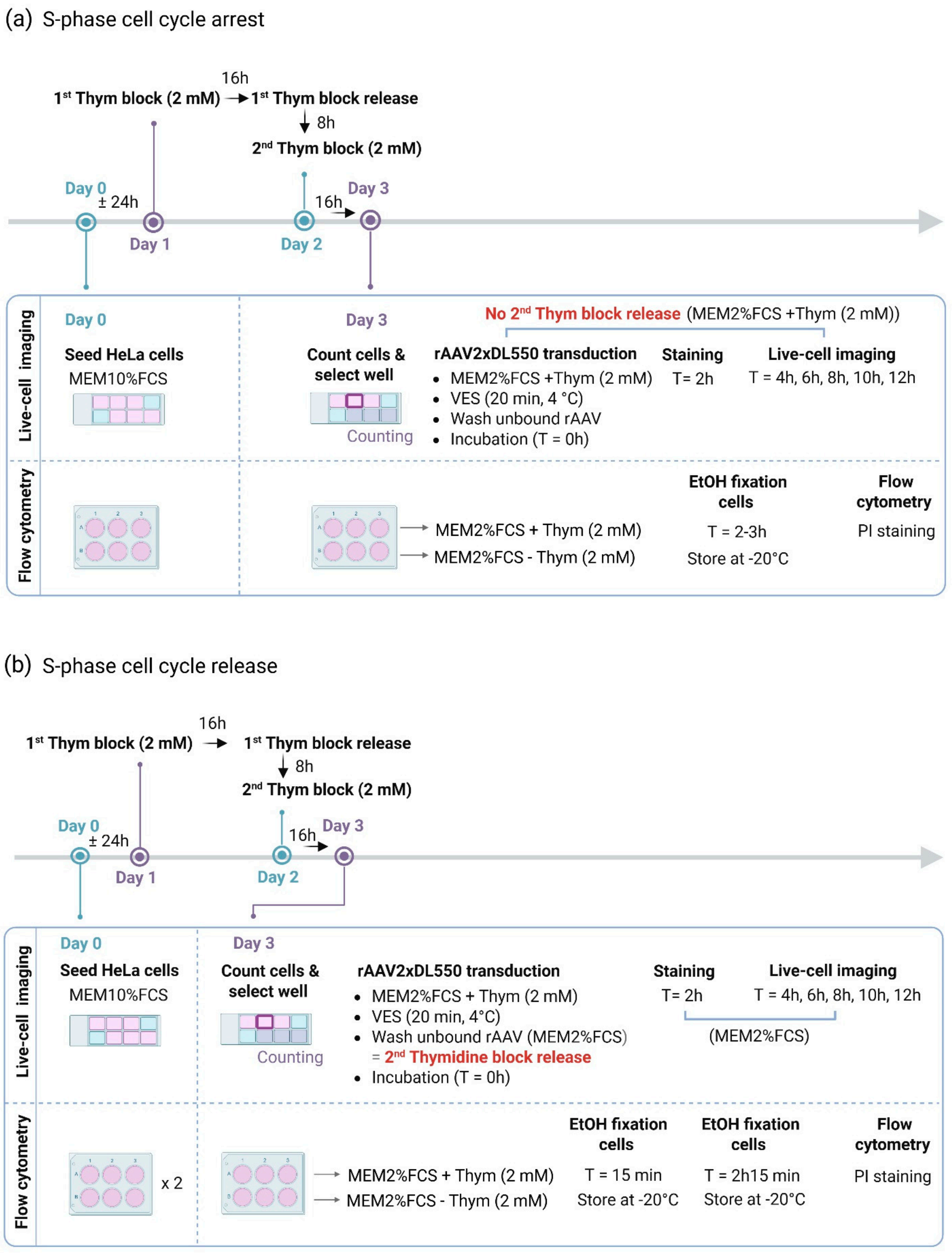

Figure S7. Live-cell imaging and flow cytometry analysis workflow to study the effect of S-phase cell cycle arrest/release on subcellular rAAV2 trafficking characteristics.

HeLa cells were seeded for live-cell imaging and flow cytometry analysis, and synchronized in S-phase using a double thymidine block (2 mM) (**a,b**). 16 Hours after the 2nd thymidine block, cells for live-cell imaging were counted, transduced, synchronized for vector entry (VES, 20 min, 4 °C), washed, and incubated. Cells were

stained with a cell plasma membrane and nuclear marker, and real-time confocal imaged from 4h until 12h post-VES all in MEM2%FCS medium containing 2 mM thymidine for continuous S-phase-arrested cells (**a**). For S-phase-released cells, unbound vector was washed, and staining and imaging was performed in MEM2%FCS without thymidine (**b**). Cells for flow cytometry analysis were seeded and synchronized in S-phase in parallel with cells for live-cell imaging, including mock treated cells that did not receive thymidine treatment. Cells were also placed at 4°C for 20 min and washed at 16 hours post 2nd thymidine block. At various timepoints post-VES (2-3h post-VES (**a**), 15 min & 2h 15 min post-VES (**b**)), cells were fixed (70% EtOH) and stored at -20°C until further PI staining-based flow cytometry analysis was performed. Three experimental repeats were performed. Thym: thymidine, MEM: Modified Eagle Medium, FCS: fetal calf serum, VES: vector entry synchronization, EtOH: Ethanol, PI: propidium iodide.

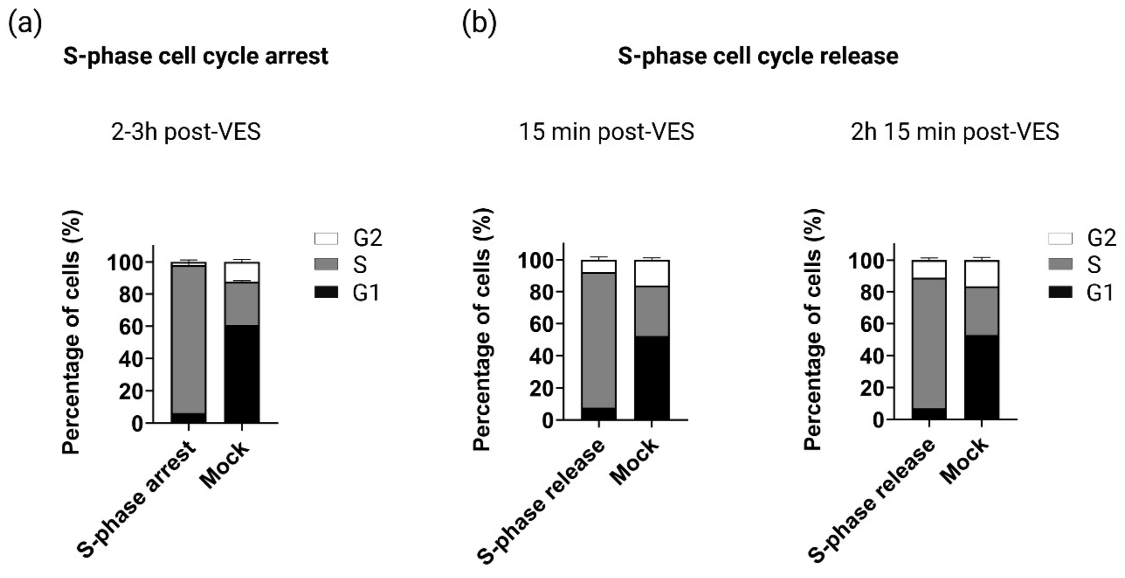

Figure S8. Effective S-phase cell cycle arrest and release in HeLa cells.
HeLa cells seeded for flow cytometry analysis were arrested in S-phase by a double thymidine block (2 mM) or mock treated. Cells were kept in or released from S-phase arrest and fixed for flow cytometry analysis at various timepoints (Figure S7). (**a**) Cell cycle profile of S-phase-arrested and mock treated cells at 2-3h post-VES. Cells were effectively blocked in S-phase as indicated by the ~3× higher percentage of cells in the S-phase (91.8%) compared to mock treated, unsynchronized cells (27.1%). (**b**) Cell cycle profile of S-phase-released and mock treated cells at 15 min and 2h 15 min post-VES. Cells were effectively released from S-phase, and gradually proceeded through the cell cycle as indicated by a decrease in percentage S-phase-blocked cells from 91.8% to 82.0%, and increasing percentage G2-phase cells from 2.0% up to 11% by 2h 15 min post-VES, compared to continuously S-phase-blocked cells (Figure S8a). Data is represented as mean ± SD from three experimental repeats. VES: vector entry synchronization.

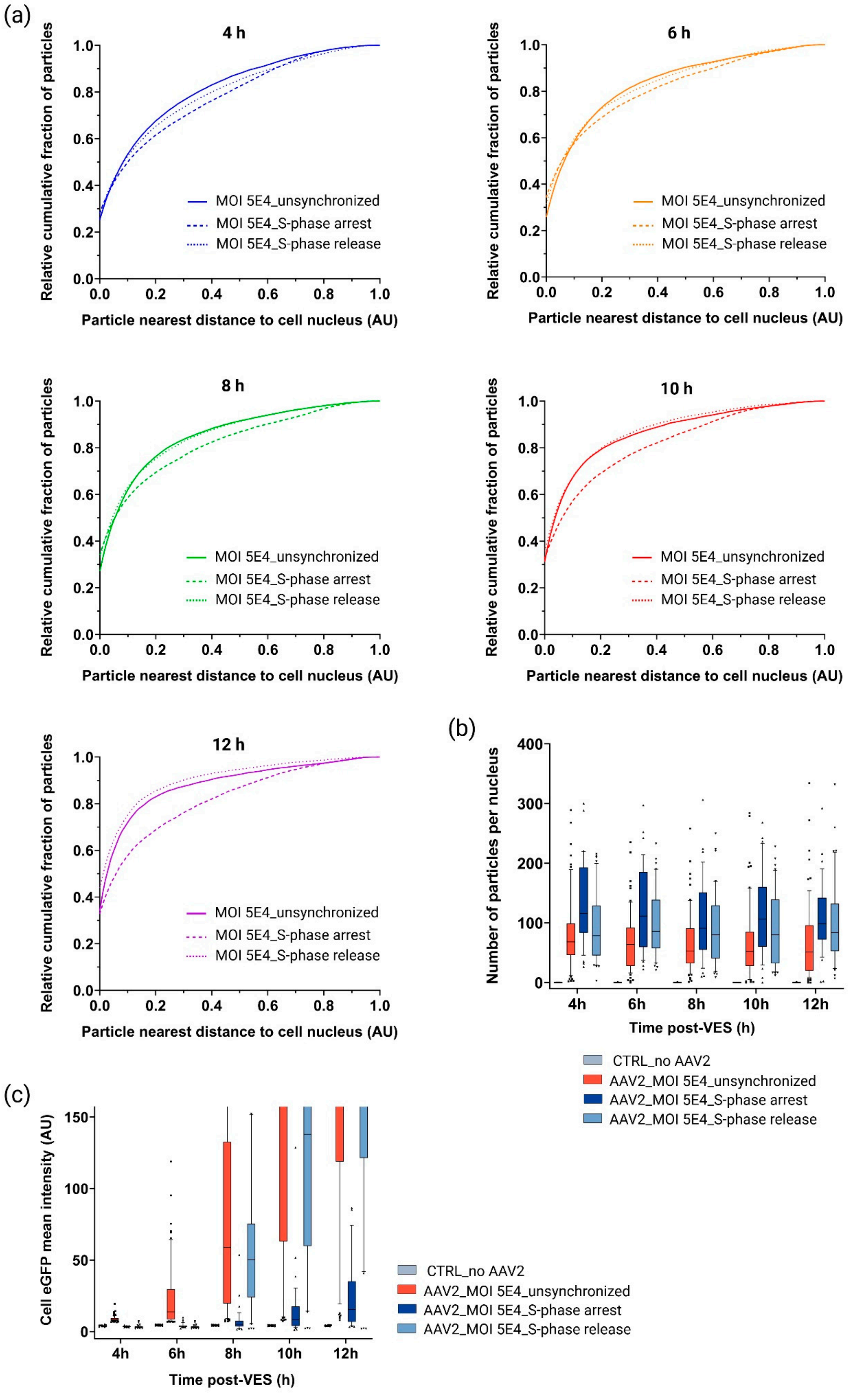

(a)
4 h
6 h
8 h
10 h
12 h
Relative cumulative fraction of particles
Particle nearest distance to cell nucleus (AU)
MOI 5E4_unsynchronized
MOI 5E4_S-phase arrest
MOI 5E4_S-phase release
(b)
Number of particles per nucleus
Time post-VES (h)
4h
6h
8h
10h
12h
CTRL_no AAV2
AAV2_MOI 5E4_unsynchronized
AAV2_MOI 5E4_S-phase arrest
AAV2_MOI 5E4_S-phase release
(c)
Cell eGFP mean intensity (AU)
Time post-VES (h)
CTRL_no AAV2
AAV2_MOI 5E4_unsynchronized
AAV2_MOI 5E4_S-phase arrest
AAV2_MOI 5E4_S-phase release

Figure S9. Spatiotemporal subcellular trafficking characteristics of unsynchronized, S-phase-arrested and S-phase-released HeLa cells transduced with DL550-labeled rAAV2.

HeLa cells were either synchronized in S-phase or unsynchronized before transduction with DL550-labeled rAAV2 (MOI 5E4), and vector entry synchronization. Transduced, unsynchronized, S-phase-arrested or S-phase-released cells were subjected to our live-cell imaging pipeline. (**a**) Cytoplasmic particle distribution in unsynchronized, S-phase-arrested and S-phase-released HeLa cells in time. Graphs represent the relative cumulative fraction of particles relative to the particle nearest distance to the cell nucleus. AU: arbitrary units, MOI: multiplicity of infection. (**b**) Image-based quantification of the number of particles per nucleus for unsynchronized (n= 67), S-phase-arrested (n=38), S-phase-released (n=40) and mock (n= 55) transduced cells per timepoint. n = number of analyzed cells per condition. (**c**) Graph with zoomed-in y axis giving a more detailed visualization of cell eGFP mean intensity for the early measured timepoints and the S-phase arrest condition (graph related to Figure 6c). Solid lines, boxes and whiskers represent medians, lower/upper quartiles and 10/90-percentile values, respectively.

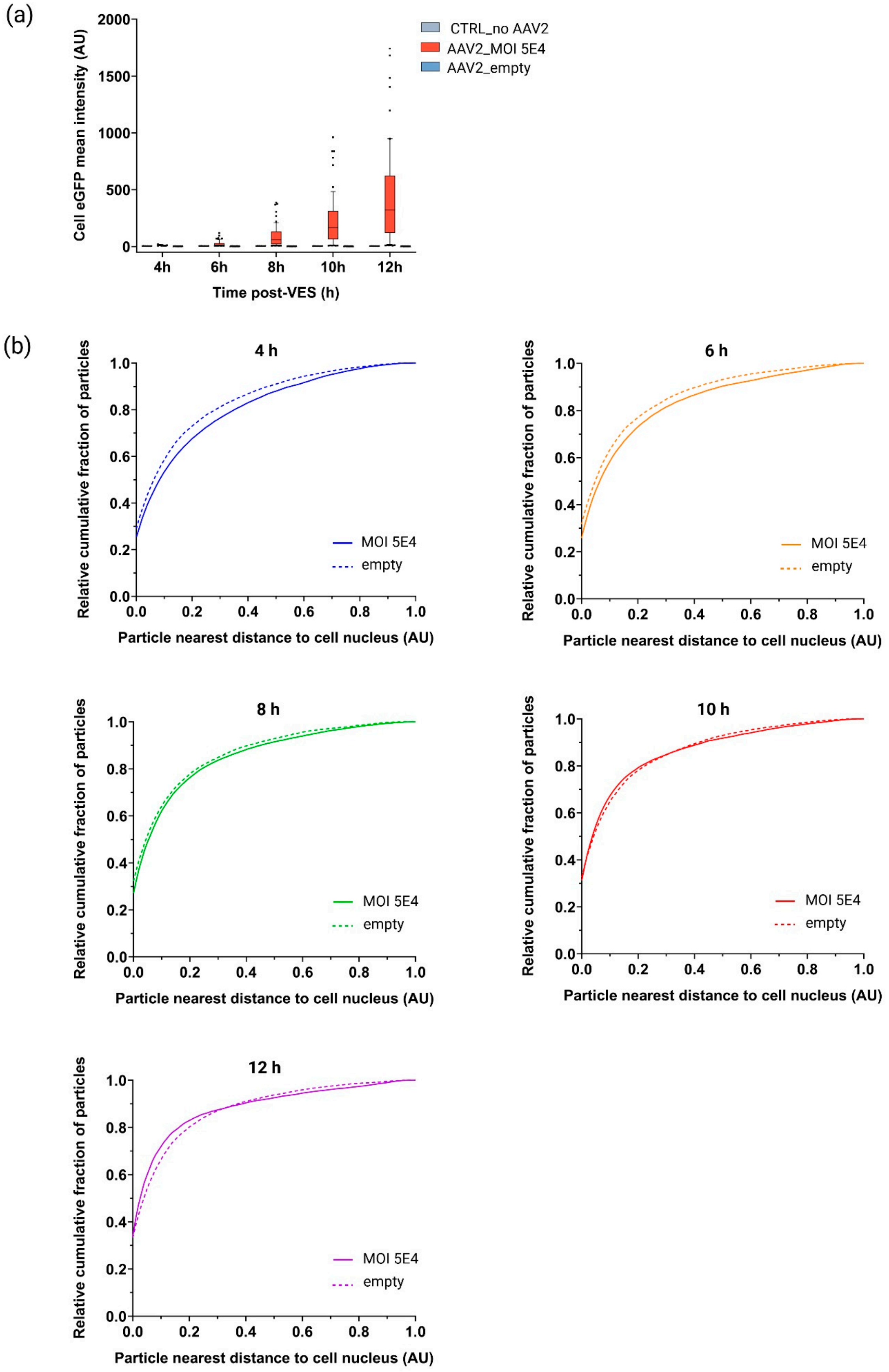
(a)
Cell eGFP mean intensity (AU)
2000
1500
1000
500
0
4h
6h
8h
10h
12h
Time post-VES (h)
CTRL_no AAV2
AAV2_MOI 5E4
AAV2_empty
(b)
4 h
6 h
8 h
10 h
12 h
Relative cumulative fraction of particles
Particle nearest distance to cell nucleus (AU)
0.0
0.2
0.4
0.6
0.8
1.0
MOI 5E4
empty

Figure S10. Spatiotemporal subcellular trafficking characteristics of empty rAAV2 particles.
(**a**) Image-based quantification of mean eGFP intensity per cell for genome-containing rAAV2 (MOI 5E4) (n= 67), empty rAAV2 (n=72), and mock (n= 55) transduced cells per timepoint. n = number of analyzed cells per condition. Solid lines, boxes and whiskers represent median, lower/upper quartile and 10/90-percentile values, respectively. (**b**) Cytoplasmic particle distribution in HeLa cells transduced with genome-containing rAAV2DL550 (MOI5E4) and empty rAAV2. Graphs represent the relative cumulative fraction of particles relative to the particle nearest distance to the cell nucleus. AU: arbitrary units, MOI: multiplicity of infection.

Time post VES	Condition	Cytoplasmic particles (%)	Perinuclear particles (%)	Nuclear particles (%)	Nuclear particles: Particles at the nuclear membrane boundary (%)	Nuclear particles: Particles completely inside nucleus (%)
4h	AAV2_MOI 5E4	72.79	4.16	23.05	8.31	14.75
	AAV2_MOI 5E3	74.33	5.46	20.21	5.72	14.49
	AAV2_MOI 5E4_S-phase arrest	70.19	4.12	25.69	9.78	15.91
	AAV2_MOI 5E4_S-pase release	70.80	4.06	25.13	10.27	14.86
	AAV2_Empty	68.89	5.43	25.69	16.61	9.07
6h	AAV2_MOI 5E4	71.56	5.61	22.83	8.45	14.38
	AAV2_MOI 5E3	75.13	4.94	19.93	7.31	12.62
	AAV2_MOI 5E4_S-phase arrest	63.87	4.60	31.53	11.27	20.26
	AAV2_MOI 5E4_S-pase release	66.48	4.53	28.99	10.18	18.81
	AAV2_Empty	65.89	6.05	28.06	17.88	10.17
8h	AAV2_MOI 5E4	70.07	5.60	24.33	8.83	15.50
	AAV2_MOI 5E3	72.46	7.75	19.79	7.43	12.36
	AAV2_MOI 5E4_S-phase arrest	63.88	4.81	31.31	12.08	19.23
	AAV2_MOI 5E4_S-pase release	64.51	4.91	30.58	10.22	20.36
	AAV2_Empty	65.23	5.67	29.10	17.36	11.75
10h	AAV2_MOI 5E4	65.63	6.26	28.11	9.62	18.50
	AAV2_MOI 5E3	67.45	8.21	24.34	8.21	16.13
	AAV2_MOI 5E4_S-phase arrest	65.72	3.87	30.41	9.93	20.48
	AAV2_MOI 5E4_S-pase release	61.77	4.86	33.37	10.28	23.09
	AAV2_Empty	64.87	5.83	29.30	17.13	12.17
12h	AAV2_MOI 5E4	62.07	7.69	30.24	10.56	19.68
	AAV2_MOI 5E3	69.33	8.57	22.10	6.48	15.62
	AAV2_MOI 5E4_S-phase arrest	65.34	4.02	30.64	9.54	21.10
	AAV2_MOI 5E4_S-pase release	53.41	5.17	41.42	12.63	28.79
	AAV2_Empty	64.17	5.60	30.23	17.07	13.16

Time post VES	Condition	Cytoplasmic particles (%)	Perinuclear particles (%)	Nuclear particles (%)	Nuclear particles: Particles at the nuclear membrane boundary (%)	Nuclear particles: Particles completely inside nucleus (%)
4h	AAV2_MOI 5E4_Non-eGFP	73.04	2.69	24.27	6.02	18.26
	AAV2_MOI 5E4_High-eGFP	72.28	4.04	23.68	8.68	15.00
6h	AAV2_MOI 5E4_Non-eGFP	76.25	3.01	20.74	7.78	12.96
	AAV2_MOI 5E4_High-eGFP	71.17	5.59	23.24	8.65	14.58
8h	AAV2_MOI 5E4_Non-eGFP	75.58	4.42	20.00	6.73	13.27
	AAV2_MOI 5E4_High-eGFP	68.08	5.69	26.23	9.59	16.64
10h	AAV2_MOI 5E4_Non-eGFP	74.06	2.71	23.23	7.55	15.68
	AAV2_MOI 5E4_High-eGFP	63.40	6.28	30.32	10.11	20.21
12h	AAV2_MOI 5E4_Non-eGFP	74.92	5.94	19.13	8.86	10.27
	AAV2_MOI 5E4_High-eGFP	59.46	7.88	32.66	10.70	21.96

Table S1. Spatiotemporal subcellular distribution of particles for all studied conditions.
Particles detected in 3D segmented HeLa cells were classified as cytoplasmic, perinuclear, and nuclear particles. Nuclear particles were further classified as particles at the nuclear membrane boundary and particles completely inside the nucleus. Classification was done based on particle distance to the cellular nucleus and nuclear ratio metrics (see Materials & Methods). VES: vector entry synchronization, MOI: multiplicity of infection.

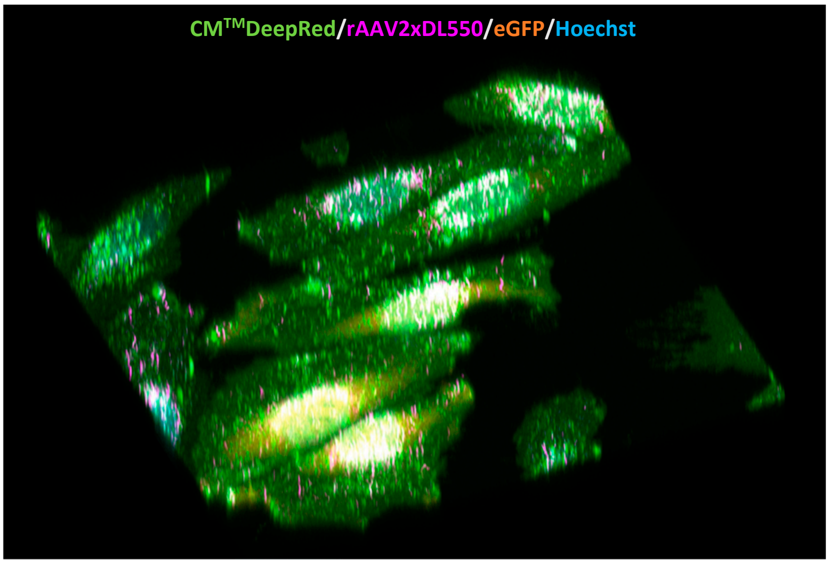

Video S1. 3D segmentation of imaged HeLa cells, nucleus and rAAV2 particles.
HeLa cells were transduced with rAAV2DL550 at MOI 5E4, synchronized for vector entry (VES, 4°C, 20 min) and incubated (37°C, 5% CO_2). Cells were stained with CellMask™ Deep Red and Hoechst to label the cell plasma membrane and nucleus, respectively at 2 hours post-VES, and afterwards imaged and analyzed using our live-cell imaging and 3D quantification platform. The video, created with the napari-animation plugin (Version 0.09) [59], shows a 3D rendered z-stack of transduced cells at 10h post-VES with cell, nuclei and viral particle segmentation. MOI: multiplicity of infection, eGFP: enhanced Green Fluorescent Protein.
